# State of inequality in malaria intervention coverage in sub-Saharan African countries

**DOI:** 10.1186/s12916-017-0948-8

**Published:** 2017-10-18

**Authors:** Katya Galactionova, Thomas A. Smith, Don de Savigny, Melissa A. Penny

**Affiliations:** 1 Department of Epidemiology and Public Health, Swiss Tropical and Public Health Institute, Socinstrasse 57, 4051 Basel, Switzerland; 2 University of Basel, Basel, Switzerland

**Keywords:** Malaria, Malaria intervention coverage, Equity, Concentration index, Asset-wealth quintiles, DHS

## Abstract

**Background:**

Scale-up of malaria interventions over the last decade have yielded a significant reduction in malaria transmission and disease burden in sub-Saharan Africa. We estimated economic gradients in the distribution of these efforts and of their impacts within and across endemic countries.

**Methods:**

Using Demographic and Health Surveys we computed equity metrics to characterize the distribution of malaria interventions in 30 endemic countries proxying economic position with an asset-wealth index. Gradients were summarized in a concentration index, tabulated against level of coverage, and compared among interventions, across countries, and against respective trends over the period 2005–2015.

**Results:**

There remain broad differences in coverage of malaria interventions and their distribution by wealth within and across countries. In most, economic gradients are lacking or favor the poorest for vector control; malaria services delivered through the formal healthcare sector are much less equitable. Scale-up of interventions in many countries improved access across the wealth continuum; in some, these efforts consistently prioritized the poorest. Expansions in control programs generally narrowed coverage gaps between economic strata; gradients persist in countries where growth was slower in the poorest quintile or where baseline inequality was large. Despite progress, malaria is consistently concentrated in the poorest, with the degree of inequality in burden far surpassing that expected given gradients in the distribution of interventions.

**Conclusions:**

Economic gradients in the distribution of interventions persist over time, limiting progress toward equity in malaria control. We found that, in countries with large baseline inequality in the distribution of interventions, even a small bias in expansion favoring the least poor yielded large gradients in intervention coverage while pro-poor growth failed to close the gap between the poorest and least poor. We demonstrated that dimensions of disadvantage compound for the poor; a lack of economic gradients in the distribution of malaria services does not translate to equity in coverage nor can it be interpreted to imply equity in distribution of risk or disease burden. Our analysis testifies to the progress made by countries in narrowing economic gradients in malaria interventions and highlights the scope for continued monitoring of programs with respect to equity.

**Electronic supplementary material:**

The online version of this article (doi:10.1186/s12916-017-0948-8) contains supplementary material, which is available to authorized users.

## Background

Over the last decade, malaria programs have been transformed with increased investment, new technologies, economic development, and shifting paradigms in global health [1]. Prior to 2008, control efforts in the region focused on groups at highest risk of adverse outcomes, especially pregnant women and children under the age of five [2]. Control programs prioritized distribution channels most likely to reach these populations such as routine vaccination campaigns, antenatal clinics, and social marketing where malaria commodities were subsidized or rendered for free [3, 4]. These channels benefitted most those with access to health facilities, which tended to be the less poor urban households [5, 6]. In 2008, a global call from the UN Secretary General placed the universal coverage of malaria interventions at the center of control efforts [7]. The aim was reflected in the Global Action Plan for Malaria [8, 9], launched the same year by the Roll Back Malaria, laying out a blueprint for control, elimination, and eventual eradication of malaria. It states that a vision of a “*malaria free world*” is brought about by “*achieving and sustaining universal access to and utilization of preventive measures; achieving universal access to case management in the public and private sectors and in the community; and accelerating the development of surveillance systems*” [9]. The aim broadened the scope of control efforts in endemic countries to include all ages and led to programs shifting from targeted distribution of insecticide-treated nets (ITNs) to mass distribution campaigns and community delivery supported by routine services. In malaria case management, fast-acting artemisinin-based combination therapies (ACTs) replaced monotherapy (‘non-artemisinin’ drug formulations) as the recommended first-line treatment for uncomplicated malaria in 2004 in response to emerging parasite resistance [10]. Initially costly and scarce, these drugs became increasingly available through the Global Fund and the novel financing mechanisms it enabled such as the Affordable Medicines Facility – malaria [11, 12]. Innovative delivery channels were introduced in many endemic countries to directly tackle distributional failures; these engaged the informal health sector and emphasized community health workers in servicing vulnerable and hard-to-reach populations [13]. As endemic countries adopted and adapted to these new policies, the malaria service gap between the poorest and the least poor began to narrow [14].

Economic gradients and progress toward equity in malaria control and curative interventions have been documented in several broad strands of literature. A large number of studies evaluated ITN coverage following a mass-distribution campaign in narrow geographic areas or within a single country showing improvement in equity after the campaign [15–18]. Socioeconomic status has also been included as one of covariates explaining coverage and use of malaria interventions; while its relative importance varied by setting and model specification, it has generally been found to be an important predictor with both use and coverage increasing with economic status [19–21]. Several multi-country analyses included ITN use among children under the age of five into a composite index for maternal and child health to track country and regional progress toward the Millennium Development Goals [22]; these studies found that, although relative inequalities have generally been narrowing, appreciable differences between the poorest and the least poor persist. There have been a number of evaluations that focused on a single malaria intervention (most commonly ITNs) [23, 24]. To the best of our knowledge, only one earlier study [5] has assessed equity across a range of malaria interventions in a large number of endemic African countries. The analysis drew on survey reports from 2006 to 2008 and, as such, was constrained to comparisons between asset-wealth quintiles. It showed that, in 13 out of 25 countries, ITN ownership was equitable while malaria treatment for febrile children and intermittent preventive treatment in pregnant women were predominantly inequitable. There has been no comprehensive assessment of malaria interventions with respect to equity since.

Monitoring equity in intervention coverage takes a new meaning today as malaria control programs mobilize efforts toward elimination [25]. Scale-up of interventions and introduction of new technologies can enhance inequities if implemented without an explicit focus on equity [26]. While wealth-related gradients have been narrowing, these gains were achieved at very low baseline coverages and with extensive donor support [27]; sustaining these gains and scaling-up toward populations at highest risk of malaria might be a challenge for many countries [28]. This study presents an update on the state of inequality in malaria intervention coverage in endemic sub-Saharan African countries through to 2015. We follow a standard framework for assessment of equity in health [29] and interpret the estimates it yields with respect to their relevance to control programs.

## Methods

### Data sources

The Demographic and Health and Malaria Indicator Surveys (DHS/MIS) are the primary sources of data for this study. Comparable in scope [30], these nationally representative household surveys collect data on a range of health behaviors and household characteristics by interviewing women of reproductive age [31]. In endemic countries, the questionnaire also covers access to, and use of, malaria interventions; in a subset of these, measurements of malaria parasite levels and anemia in a sample of children and pregnant women are included [32]. In the primary analysis, we included countries that conducted at least one survey between 2011 and 2016; of these, countries with repeated surveys between 2003 and 2016 were included in trend analyses. Surveys by country are listed in Additional file 1.

In an auxiliary analysis we additionally used 2015-modeled surfaces of malaria endemicity (mean *P. falciparum* prevalence in children aged between two and ten (*Pf*PR_2-10_)) from the Malaria Atlas Project [33] to characterize malaria transmission at sub-national level. This was accomplished by mapping GPS locations of survey clusters into malaria endemicity surfaces [30, 34]; *Pf*PR_2-10_ values at the centroid coordinates of the cluster were assigned to all households.

To enhance graphical representation of estimates we also merged in the 2015 country mean *Pf*PR_2-10_ from the Malaria Atlas Project and population estimates from the World Bank [35].

### Outcomes

We assessed intervention coverage with a subset of standard monitoring and evaluation indicators adopted in global malaria strategic documents and endorsed by endemic countries to track progress toward control and elimination targets [19–21]. The indicators selected for the analysis relate most directly to malaria outcomes (protection or cure). For instance, we focus on adequate access to ITNs within households covering all members and compliant malaria prophylaxis for pregnant women during an antenatal care visit rather than the broader indicators of household access to any ITN or receipt of at least one dose of sulfadoxine-pyrimethamine during pregnancy. These more stringent definitions will yield lower coverages and potentially higher inequality than estimates based on indicators defined more broadly.

We assessed six malaria intervention coverage indicators (‘coverage’ is used loosely herein to cover both access and use of interventions). To capture the reach of national malaria control programs, we evaluated ITN coverage as a proportion of households with at least one ITN for every two persons. ITN use was estimated as the proportion of population that slept under an ITN the previous night. Coverage of insecticide residual spraying (IRS) was expressed as a proportion of households residing in dwellings sprayed with an insecticide within the last 12 months. This definition is broader than the population targeted by the intervention. Unlike other malaria interventions that are deployed nationally, IRS is implemented only in foci of high malaria transmission in a subset of countries in the region. Constrained by the data, malaria prophylaxis was evaluated only among women; we calculated the proportion of recent mothers (live birth within last 2 years) that received at least three doses of sulfadoxine-pyrimethamine during antenatal care visits. The distribution of curative interventions was estimated among children under the age of five and conditional on fever. Despite progress, access to ACTs (current WHO recommended first-line treatment for uncomplicated malaria [36]) remains low, with many antimalarial drugs other than the country recommended first-line medication in wide circulation [34, 37]. To allow for a broader snapshot of malaria case management, we included two indicators, namely the proportion of children with fever that sought care at a formal provider (i.e., outpatient department, inpatient department, government and private health centers), and the proportion of children with fever that were treated with any antimalarial medication, including both ACT and non-ACT antimalarials. More narrow definitions of malaria case management were also assessed, including the proportion of children with fever that received the first-line antimalarial according to country policy, and are reported in Additional files; we refer to these estimates when discussing malaria treatment.

Of health indicators, we evaluated malaria parasitaemia in children aged 6–59 months according to microscopy. Given the lack of a gold standard diagnostic test for malaria that is practical for use in national survey settings [32], we also report prevalence according to rapid diagnostic tests (RDT). RDT parasite prevalence estimates are generally higher and show somewhat stronger economic gradients than those based on microscopy.

### Socioeconomic status stratifier

Distribution of malaria intervention coverage and health indicators was evaluated against an asset-wealth index; this stratifier reflects the relative economic standing of households in a given country at the time of the survey [38]. Derived from a national distribution of assets weighted with principal component analysis and adjusted for place of residence the index has been shown to perform well in identifying the most disadvantaged groups akin to other measures of relative poverty. It has been used extensively in health equity research in low-income settings including for malaria [5, 22, 24, 39, 40].

### Statistical analysis

We calculated absolute and relative differences in indicators between households in lowest and highest wealth quintiles. We also assessed the degree of inequality in indicators across the whole distribution of asset-wealth scores with a concentration index (CIX). The CIX is a relative measure of inequality that indicates the extent to which an indicator is concentrated among the disadvantaged or the advantaged within a particular setting. It is defined as twice the area between the concentration curve and the line of equality [41]. Negative values of CIX denote a disproportionate concentration of service or health variables among the poorest, positive values - among the least poor. The index and the corresponding 95% confidence intervals were computed with the *conindex* command in Stata 14 SE [42]. Changes in coverage over time were expressed in terms of excess change [29]. This metric represents the difference in average annual change in a given indicator between the lowest and highest asset-wealth quintiles. Considered along the absolute change in population average the metric allows categorization of the trend in outcome with respect to equity. A negative excess change at an increasing population average suggests the expansion is pro-poor (higher average annual increase in the poorest quintile compared to the least poor), and so forth [43]. We refer to distributions or changes in distribution that favor the least poor as ‘inequitable’, while inequalities and changes that favor the poor are termed ‘pro-poor’ [29].

The indicators were coded following the Household Survey Indicators for Malaria Control [44] and replicate DHS estimates in final country reports [45]. All reported statistics are population weighted and account for the complex survey design. The analysis was implemented in Stata 14 SE [46].

## Results

Overall, across countries and coverage indicators, malaria programs in the region are inequitable (Table 1). Yet, in 16 out of 30 countries, ITN ownership is distributed equally or favors the poorest; in over half of the countries, ITN use and IRS are distributed equally or favor the poorest; for interventions relying on the formal health sector, such as intermittent preventive treatment in pregnancy (IPTp) and access to formal care, provider inequities are not evident in nearly half of the countries. These categorizations refer to relative differences in coverage of malaria interventions across the asset-wealth continuum within countries and do not take into account the level of intervention. For instance, in Benin, where household access to at least one ITN for every two persons is disproportionately higher among the least poor (CIX = 0.07), the poorest strata have better access to ITNs in absolute terms than the poorest strata in Cote d’Ivoire, where the indicator is equitably distributed (42% and 34%, respectively) (Additional file 2: Table SA3).

**Table 1:** Summary of distribution of malaria interventions across asset-wealth index in sub-Saharan African countries in 2015^a^

Intervention	Pro-Poor	Inequitable	No difference across the asset-wealth index distribution
Household with at least one insecticide-treated net (ITN) for every two persons	CIV, COG, GAB, GHA, LBR, NAM, ZWE	AGO, BDI, BEN, BFA, CMR, COM, KEN, MLI, MOZ, MWI, NER, RWA, TZA, ZMB	COD, GIN, MDG, NGA, SEN, SLE, TCD, TGO, UGA
Population who slept under an ITN last night	CIV, COG, GAB, GHA, GIN, LBR, MDG, NAM, SLE, TGO, UGA, ZWE	AGO, BDI, CMR, COM, KEN, MOZ, MWI, NER, RWA, TCD, TZA	BEN, BFA, COD, MLI, NGA, SEN, ZMB
Household with insecticide residual spraying in the past 12 months	BDI, BEN, COM, GHA, LBR, NAM, SEN, UGA, ZWE	BFA, CIV, CMR, GAB, GIN, MOZ, NER, SLE, TCD, ZMB	MDG, MLI, MWI, NGA, TZA
Mother received 3+ doses of sulfadoxine-pyrimethamine during antenatal care visit		AGO, BEN, CIV, CMR, GHA, GIN, MLI, MOZ, NER, NGA, TCD, TGO, TZA, ZMB	BDI, COD, COG, COM, GAB, KEN, LBR, MDG, MWI, NAM, SEN, SLE, UGA
Child (<5 years) with fever sought care at a formal provider		AGO, BEN, BFA, CIV, CMR, COG, GIN, KEN, LBR, MLI, MOZ, NER, NGA, RWA, TCD, TGO, TZA, UGA, ZWE	BDI, COD, COM, GAB, GHA, MDG, MWI, NAM, SEN, SLE, ZMB
Child (<5 years) with fever treated with an antimalarial	MOZ, MWI, TZA, ZMB	AGO, BEN, BFA, CIV, CMR, COD, GAB, GIN, NER, NGA, TCD	BDI, COG, COM, GHA, KEN, LBR, MDG, MLI, NAM, RWA, SEN, SLE, TGO, UGA, ZWE

For each country (indicated by ISO3 codes), distribution of malaria intervention coverage indicators were assessed over population ranked by asset-wealth; the degree of inequality in distribution of interventions was summarized in concentration index (CIX). Interventions were classified as “Pro-poor” if the estimated CIX was negative, “Inequitable” if was CIX is positive, and as “No difference across the asset-wealth index distribution” if CIX was equal to zero or lacked statistical significance

^a^Data drawn from a subset of countries with Demographic and Health Survey/Malaria Indicator Survey conducted after 2010 (ISO3 codes and years of data collection are detailed in Additional file 1: Table SA1)

**Fig. 1:**
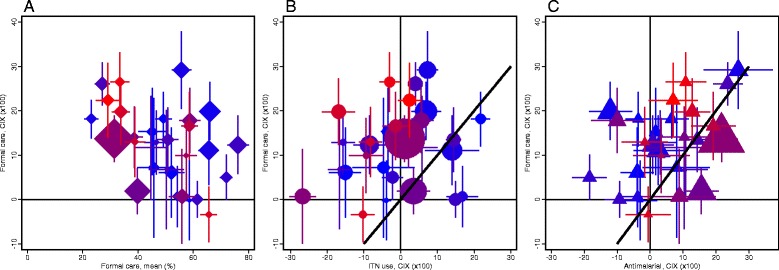
Degree of asset-wealth inequality in distribution of malaria interventions in sub-Saharan African countries in 2015^a^. For each country, the concentration index (CIX) is plotted for (**a**) access to formal care for fever among children under the age of five is plotted against its population mean (proportion), (**b**) ITN use, and (**c**) receipt of antimalarial medication for children under the age of five with fever. Whiskers denote the 95% confidence interval of the estimate. Country marker size is weighted with population size. Marker color code changing from bright blue to bright red refers to country mean malaria prevalence based on 2015 Malaria Atlas Project estimates (corresponding values are given in Additional file 1: Table SA1). ^a^Data drawn from a subset of countries with Demographic and Health Survey/Malaria Indicator Survey conducted after 2010 (country list and year of data collection are detailed in Additional file 1: Table SA1).

Figure 1 illustrates the strength of economic gradients in distribution of malaria interventions qualified above, thereby addressing the question of the existing level of inequality, comparing this across interventions. The CIX for access to formal care – the most consistently inequitable coverage indicator – is plotted against country mean (Fig. 1a). Inequity is highest in settings with lowest access. Most high *Pf*PR_2-10_ countries (in shades of red) are at this end of the distribution; Ghana is one notable exception. For all other intervention coverage indicators, we found little correlation between level and degree of inequality (Additional file 3); that is, higher coverage is not necessarily described by a more equitable distribution nor is low coverage consistently inequitable.

Compared to access to formal care, ITN use is distributed more equitably (most country estimates above the equality diagonal) (Fig. 1b). High *Pf*PR_2-10_ countries with large inequities in access to formal care (positive values of concentration index on y-axis) appear to be equitable or pro-poor in distribution of ITN use (clustering of points around zero and –10 values of rescaled CIX on x-axis). In Congo, Democratic Republic of the Congo, Togo, and Uganda, access to formal care strongly favors the least poor, while ITN use favors the poorest. On the opposite end of the plane are a handful of lower *Pf*PR_2-10_ countries, including Burundi and Malawi, where inequality in ITN use exceeds that in access to formal care.

Anti-malarial treatment is equitable or slightly pro-poor in low *Pf*PR_2-10_ countries (shown in shades of blue) (Fig. 1c). In high *Pf*PR_2-10_ countries, antimalarial treatment favors the least poor, though the degree of inequality is lower than that estimated for formal care. There are a few exceptions to this general trend. In Democratic Republic of the Congo and Nigeria, both medium *Pf*PR_2-10_ countries, the distribution of antimalarial treatment is more inequitable than that of formal care. In Togo, a high *Pf*PR_2-10_ country, antimalarial treatment is distributed equitably despite large gradients in formal care favoring the less poor. Distribution of first-line medication is consistent with that of receipt of any antimalarial described above (Additional file 3: Figure SA2). The two indicators are correlated most closely in settings with lower *Pf*PR_2-10_, where first-line drugs account for a large fraction of antimalarials dispensed. In higher *Pf*PR_2-10_ countries, inequities in first-line treatment are lower than those estimated for any antimalarial medication; however, access to these medications is also significantly lower. For instance, in Nigeria, about 33% of children with fever are treated with an antimalarial, while only about 6% receive the first-line drug; the least poor are more than twice as likely as the poorest to receive either of the medications (Additional file 2: Tables SA8-SA9).

**Fig. 2:**
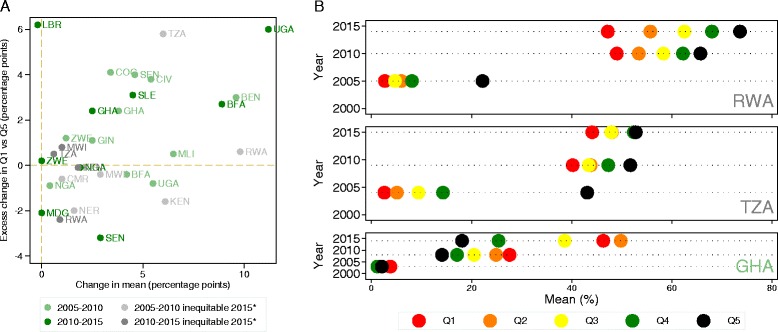
Changes in distribution of ITN use by asset-wealth in sub-Saharan African countries from 2005 to 2015*. **a** The difference in average annual change for each country in the proportion of population that slept under an ITN the night prior to the survey in the lowest asset-wealth quintile and that of the highest (annual absolute excess change) against average annual change in the population (percentage points). **b** The proportion of the population for each of the DHS surveys that slept under an ITN the night prior to the survey in each of the five wealth quintiles (Q1–Q5) for Rwanda (RWA), Tanzania (TZA), and Ghana (GHA). Data drawn from a subset of countries with repeated Demographic and Health Survey/Malaria Indicator Survey conducted between 2005 and 2015 (country list, ISO3 code, and years of data collection detailed in Additional file 1: Table SA2).

ITN use is the most equitably distributed malaria intervention coverage indicator. Figure 2 illustrates changes in its distribution over the last decade. ITN use expanded in all countries (positive values of annual absolute change on the x-axis) between 2005 and 2015 (Fig. 2a); it increased somewhat faster between 2005 and 2010 compared to the later period. In most countries, these expansions were pro-poor (positive values of excess change on the y-axis). Where expansions favored the least poor (negative values of excess change on the y-axis), the relative gains in coverage were smaller in absolute terms than the excess change in settings where growth was pro-poor (positive values of excess change on the y-axis). It is primarily in these countries where interventions expanded faster in the least poor strata where we see inequities in distribution of ITN use today (in shades of grey). Figure 2b presents a more nuanced picture of intervention expansion path in three countries, showing that failure to sustain a pro-poor focus in expansion yields greater inequality in later periods (Rwanda), that high baseline inequality in service coverage defies pro-poor growth (Tanzania), and that, in some countries, expansion in ITN use consistently prioritized the poorest strata (Ghana).

While relatively smaller, improvements in coverage occurred across all interventions delivered by malaria control programs (Additional file 4). In about half of the countries, these expansions were pro-poor or distributed equally across asset-wealth strata for ITN ownership and access to formal care. Changes in IPTp were mainly driven by improvements in the least poor strata from 2005 to 2010, with somewhat more equitable growth in the later period. Interpretation of changes in use of antimalarial medication with respect to program performance is confounded by changes in malaria epidemiology and the shift toward diagnostically confirmed treatment. Overall use of antimalarials decreased in most countries with a relatively lower reduction in the least poor compared to the poorest strata. There is limited evidence that the direction of change reversed toward expansion in the poorest strata in the most recent period.

By pursuing malaria intervention coverage targets, countries aim at a broader health and health equity agenda. Tabulating malaria prevalence for a subset of DHS/MIS countries that included malaria diagnosis, we found that parasitaemia remains disproportionally concentrated in the poorest; prevalence gradients are substantially higher than what might have been expected given distribution of coverage indicators (Table 2). Even in countries where malaria interventions are equitable or favor the poor (i.e., Ghana) differences in burden between the poorest and least poor are large.

**Table 2:** Distribution of malaria prevalence across asset-wealth quintiles in sub-Saharan African countries in 2015^a^

Country	Total	Poorest quintile Q1	Richest quintile Q5	Difference Q5-Q1	Ratio Q5:Q1	Concentration index (×100)
Benin	28.5 (26.6 to 30.4)	39.6 (35.2 to 44.0)	15.4 (12.0 to 18.9)	–24.1 (–29.8 to –18.5)	39.0 (29.2 to 48.8)	–21.0 (–25.3 to –16.8)
Burkina Faso	45.9 (43.1 to 48.7)	57.9 (53.1 to 62.7)	13.9 (8.8 to 19.1)	–44.0 (–51.0 to –36.9)	24.1 (14.9 to 33.2)	–32.8 (–37.6 to –28.0)
Burundi	17.3 (13.6 to 21.0)	25.9 (19.6 to 32.2)	8.4 (4.7 to 12.1)	–17.5 (–24.4 to –10.6)	32.4 (16.8 to 48.0)	–14.3 (–19.7 to –9.0)
Congo, Democratic Republic	22.6 (20.4 to 24.8)	23.0 (19.6 to 26.5)	12.4 (9.2 to 15.6)	–10.6 (–15.4 to –5.9)	54.0 (37.8 to 70.1)	–7.7 (–11.8 to –3.6)
Cote d’Ivoire	18.0 (15.3 to 20.8)	28.2 (22.8 to 33.6)	4.3 (1.8 to 6.7)	–24.0 (–29.9 to –18.0)	15.1 (6.0 to 24.1)	–20.6 (–25.8 to –15.5)
Ghana	26.7 (23.9 to 29.6)	42.1 (36.0 to 48.3)	7.5 (3.9 to 11.2)	–34.6 (–41.8 to –27.5)	17.9 (8.9 to 26.8)	–30.8 (–36.3 to –25.2)
Guinea	43.9 (40.5 to 47.2)	56.9 (51.7 to 62.2)	8.3 (4.0 to 12.6)	–48.6 (–55.5 to –41.8)	14.6 (6.9 to 22.3)	–32.1 (–38.2 to –26.1)
Kenya	5.0 (3.7 to 6.2)	5.8 (3.2 to 8.4)	0.9 (–0.2 to 1.9)	–4.9 (–7.7 to –2.1)	15.3 (–4.2 to 34.8)	–4.1 (–6.5 to –1.7)
Madagascar	6.9 (4.8 to 9.0)	14.1 (9.8 to 18.4)	0.4 (–0.1 to 0.9)	–13.7 (–18.1 to –9.4)	2.6 (–1.1 to 6.3)	–11.6 (–15.2 to –7.9)
Mali	51.6 (48.7 to 54.5)	69.4 (65.4 to 73.4)	15.1 (11.8 to 18.3)	–54.3 (–59.5 to –49.1)	21.7 (16.8 to 26.6)	–41.6 (–46.2 to –37.1)
Mozambique	35.1 (32.0 to 38.2)	53.3 (47.8 to 58.7)	6.5 (4.2 to 8.8)	–46.8 (–52.5 to –41.0)	12.2 (7.8 to 16.6)	–37.7 (–42.7 to –32.7)
Rwanda	2.2 (1.6 to 2.9)	4.7 (3.1 to 6.3)	0.2 (–0.2 to 0.5)	–4.5 (–6.2 to –2.8)	3.9 (–3.9 to 11.7)	–3.7 (–5.1 to –2.3)
Senegal	1.2 (0.6 to 1.8)	3.5 (1.6 to 5.4)	0.4 (–0.4 to 1.3)	–3.1 (–5.2 to –0.9)	12.4 (–13.8 to 38.6)	–2.7 (–4.4 to –1.1)
Tanzania	5.6 (4.6 to 6.5)	8.0 (6.0 to 10.0)	1.0 (0.4 to 1.6)	–7.0 (–9.1 to –4.9)	12.2 (3.9 to 20.5)	–6.8 (–8.6 to –4.9)
Togo	36.4 (33.3 to 39.5)	49.1 (44.1 to 54.0)	9.2 (6.0 to 12.4)	–39.8 (–45.7 to –33.9)	18.8 (12.1 to 25.6)	–35.2 (–40.2 to –30.2)
Uganda	20.1 (17.2 to 22.9)	29.8 (24.5 to 35.1)	4.0 (2.1 to 5.9)	–25.8 (–31.6 to –20.1)	13.3 (6.4 to 20.3)	–19.4 (–24.0 to –14.9)

Microscopy-confirmed malaria prevalence (percent) in children aged 6–59 months assessed in a representative sub-sample of the Demographic and Health Survey/Malaria Indicator Survey (DHS/MIS) surveyed population. 95% confidence intervals adjusted for survey design are reported in parenthesis. Prevalence estimates according to rapid diagnostic tests are reported in Additional file 5

^a^Data drawn from a subset of countries with Demographic and Health Survey/Malaria Indicator Survey conducted after 2010 (ISO3 codes and years of data collection are detailed in Additional file 1: Table SA1)

## Discussion

Decisive strides against malaria over the last decade have enabled significant expansion of control programs across endemic countries [47]; in some, these efforts also achieved equity in intervention coverage [5, 22, 24, 48, 49]. More recent data analyzed here show that, not only has the number of countries with equitable or pro-poor ITN ownership and use increased by 2015, but also that the degree of inequality in countries where inequities persisted is modest with the exception of a handful of low *Pf*PR_2-10_ settings. Large wealth-related gaps previously shown in access to antimalarial medication [5] narrowed, with 19 out of 30 countries reporting equitable or pro-poor coverage. These include most Affordable Medicines Facility – malaria pilot phase countries, except Niger and Nigeria, where access to antimalarial medication remains substantially inequitable. Important gains have also been made in delivering IPTp to poor women – in 13 out of 27 countries, this preventive intervention is equitable. In Namibia and Senegal, all malaria control interventions are distributed equitably; whereas in Ghana and Liberia and in Sierra Leone, all control interventions, except for IPTp and IRS, respectively, are distributed equally.

Where asset-wealth differences in coverage persist, the relative performance of malaria interventions with respect to equity both within and between countries provides useful benchmarks. The large economic gradients in the distribution of curative interventions across countries highlight the difficulty of delivering routine care to the poor, but differences in relative degree of inequality (i.e., a highly inequitable CIX of 0.27 in distribution of access to formal care in Guinea compared to a CIX of 0.02 in Democratic Republic of the Congo) suggest that it is feasible to substantially reduce these inequities. Benin and Mali achieve coverage and use of ITNs at rates that are approximately 20 and 40 percentage points above those estimated for formal care; in these countries, ITN use is also distributed equitably across asset-wealth strata in contrast to large economic gradients in curative care. The differential performance of interventions within control programs indicates that there is technical capacity to identify and deliver services to the poor; what is needed is implementation insight to transfer efficiency of high-performing interventions to those lagging behind [50]. Review of malaria programs covering health systems, financing, and operational contexts is needed to gather and synthesize institutional expertise from within the region.

Differences in the level and distribution of malaria interventions within endemic countries also impact adoption, prioritization, and deployment of new malaria tools. Our analyses suggest that, in high endemic settings, interventions deployed via campaigns are likely to be more equitably distributed than those requiring routine delivery through the formal sector. These considerations are immediately relevant for countries currently considering introduction of new malaria interventions such as malaria vaccines [51]. The economic gradients estimated here for access to the formal sector are representative of the EPI coverage in endemic settings [52], suggesting that much of the vaccine’s impact is likely to be missed due to distributional failures unless routine immunization is supplemented with periodic intensification and outreach campaigns.

In many high *Pf*PR_2-10_ countries, coverage of malaria interventions remains low; how programs scale-up has important implications for equity [24], in agreement with a previous analysis [20] showing that, where ITN use increased, gains were made across the asset-wealth continuum, often with accelerated growth in the poorest stratum. We find this conclusion also holds for other malaria coverage indicators, indicating that expansions in malaria programs in the region have largely circumvented the inverse care law [51]. Yet, despite pro-poor growth, progress toward equitable distribution of malaria interventions in many settings is limited by past high levels of inequality; turning these programs around is a challenge. For these countries, failure to sustain a focus on equity in service delivery leads to greater inequality in subsequent periods, while narrowing the gap between poorest and least poor requires disproportionately faster and sustained growth in the poorest strata.

These dynamics can partially explain systematic differences in the level and distribution of malaria coverage indicators by *Pf*PR_2-10_. Most high *Pf*PR_2-10_ countries were late to scale-up ITNs; these efforts relied heavily on donor assistance that financed mass distribution campaigns outside the formal health sector, enabling equitable coverage in these settings. In lower *Pf*PR_2-10_ countries, ITN delivery relied more on routine channels propagating disparities in access to formal care to ITN coverage. Distributional failures in the formal health sector limit progress toward equity across malaria interventions as programs rely on routine channels to maintain coverage between campaign rounds (i.e., ITN, IPTp), to strengthen access for priority groups, and to inform planning and resource allocation decisions with data sourced from governmental health facilities. Tackling malaria requires a holistic view of the program; to succeed, there should be a focus on strengthening the health systems at large.

**Fig. 3:**
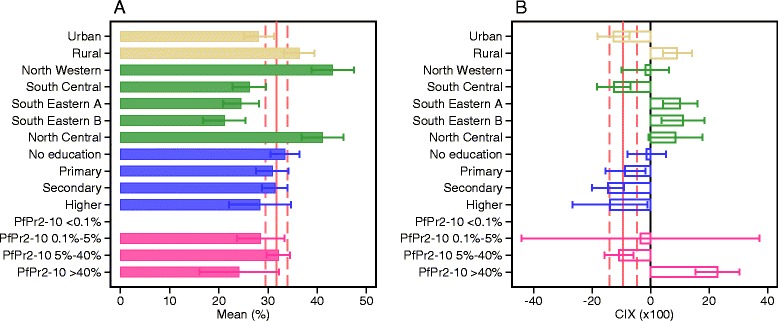
Heterogeneity in distribution of malaria interventions over transmission and alternate socioeconomic stratifiers in Liberia in 2015^a^: proportion of population who slept under an ITN the previous night. **a** Mean and 95% confidence interval for the proportion of population who slept under an ITN the previous night within each dimension: place of residence (urban or rural), region ((North Western, South Central, South Eastern A, South Eastern B, North Central), mother’s highest educational level (no education, primary, secondary, higher), *Pf*PR_2-10_ level (<0.1%, 0.1–5%, 5–40%, > 40%). The national average and the corresponding confidence interval are shown in solid and dash pink lines respectively. **b** The degree of inequality, summarized here in a concentration index and 95% confidence interval, in distribution of ITN use with respect to asset-wealth index within each dimension. National average of the concentration index and the corresponding confidence interval are shown in solid and dash pink lines respectively. ^a^Data drawn from Liberia Demographic and Health Survey 2013

Evidence of economic gradients in intervention coverage lends itself to a clear programmatic interpretation – the poor are left behind; but analogous interpretation of equitable or pro-poor gradients may be misleading. First, as dimensions of social and economic disadvantage overlap, analyses limited to broad comparisons between the poorest and the least poor might average over important gradients understating the true degree of disparity [53]. Second, it is not obvious whether gradients defined over an asset-wealth index capture policy-relevant dimensions of economic or social disadvantage in malaria-endemic countries [39, 40, 54]. Finally, geographic and demographic clustering of malaria in low transmission settings might undermine the capacity of conventional tools to identify and monitor high-risk populations [25]. To illustrate, we analyzed ITN use by a number of common equity stratifiers and an interaction of these dimensions with the asset-wealth index for Liberia, a country with a pro-poor or egalitarian distribution of most malaria interventions (Fig. 3). Here, ITN use is equally distributed across the economic strata at the national level (solid and dashed pink lines); it is effectively equal to national average when stratified by place of residence, education, or *Pf*PR_2-10_ (colored bars) (Fig. 3a). However, large gradients for Liberia emerge when either of the dimensions is overlapped with the asset-wealth index (Fig. 3a). There are large regional differences around the national average and even stronger economic gradients within these regions. While pro-poor at national level, ITN use is concentrated among the least poor in rural settings, areas of high *Pf*PR_2-10_, and in all regions except for the South Central region of Liberia, which includes the country capital.

Equality across wealth quintiles in coverage, or concentration of interventions in the poorest, is likely to be more effective in reducing burden than concentration of interventions in the richer quintiles because of the association of malaria infection with low socioeconomic status (Table 2) [55]. The areas and individuals at highest risk contribute disproportionately to onward infection [56], so targeting them is highly effective if they can be identified [57]. However, identifying them is challenging [58]. Economic and social dimensions have a role in this, and thus need to be considered as important components (alongside epidemiological and geographic data) in new approaches in malaria mapping, in the development of more comprehensive measures of malaria vulnerability [59, 60], and in analytical tools to help malaria control programs direct efforts towards vulnerable sub-populations [61, 62].

By eliminating disparities in proximal determinants (access to preventive and curative interventions) of malaria morbidity and mortality, control programs in endemic countries address only a part of the causal pathway from exposure to disease; the underlying socioecological conditions could manifest through other mechanisms further perpetuating health inequities [58]. The consideration is relevant to evaluation and performance targets for control programs. Differential tracking of coverage indicators by malaria endemicity and/or other context-specific dimensions of social or economic disadvantage, as shown here, can enhance equity analyses [28, 32] by focusing most directly on populations at risk. Expanding the scope of monitoring indicators to also include quality dimensions could help direct efforts toward increasing efficiency of current control tools. To this end, the equity effectiveness framework can be applied to disentangle the impact of various factors on the gap in the effectiveness of interventions across socioeconomic gradients [52]. For instance, compounding of disadvantage along the service delivery path has been shown for malaria case management, where the poor are not only less likely to seek care but also less likely to do so in the formal sector, or to receive appropriate diagnosis and treatment [32].

There are a number of limitations to consider when interpreting our findings. Due to varying survey cycles between countries, there is some inconsistency in reference years for reported statistics. To the extent that expansion in malaria services accelerated closer to 2015 or accelerated differently with respect to equity, estimates based on less recent surveys understate the level of service provision, overstate the degree of inequality in their distribution across the wealth strata, and overstate differences in the two statistics between countries. Timing of campaigns (i.e., IRS or ITN distribution) with respect to survey implementation is another variable affecting the estimated level of malaria services and comparisons thereof between countries. Finally, by design, DHS surveys are to be conducted during low transmission season, while MIS is conducted at peak. Survey implementation in the field, however, spans months averaging over the seasonality pattern in the data collected [30]. To the extent that in some countries the pattern is maintained comparisons will be biased.

## Conclusions

The global health and development agenda place a special emphasis on universal health coverage [63–65]. The Global Technical Strategy for Malaria 2016–2030, in particular, argues for universal access to interventions for malaria prevention, diagnosis, and treatment as a path toward elimination [63]. For endemic countries, given the historically low access among the poorest, adopting this vision would require overall gains in coverage to be accompanied with accelerated improvements in disadvantaged populations. Our analysis testifies to the progress made by countries in the region in narrowing economic gradients in malaria intervention coverage and highlights the scope for continued monitoring of these programs with respect to equity.

## Additional files

Additional file 1: Tables SA1. and **SA2** that list by country: country name, ISO3 code, mean population weighted *PfPR*_*2-10*_, and corresponding DHS/MIS survey used in 2015 tabulations and 2005–2015 year trend analyses. (DOCX 37 kb)

Additional file 2: Tables detailing country estimates corresponding to level and degree of inequality for each of the malaria intervention coverage indicators including ACTs (Tables SA3-SA9). (DOCX 93 kb)

Additional file 3: Plots of 2015* level and degree of inequality for each of the malaria intervention coverage indicators including ACTs (Figure SA1-SA2). (DOCX 84 kb)

Additional file 4: Plots of excess change and change in each of the malaria intervention coverage indicators from 2005 to 2015* (Additional file 2: Figure SA2). (DOCX 65 kb)

Additional file 5: Table SA10. 2015* malaria parasite prevalence estimates according to rapid diagnostic tests based on country DHS/MIS surveys. (DOC 45 kb)

## Abbreviations

ACT: artermisinin combination therapy
CIX: concentration index
DHS: Demographic and Health Survey
IPT: intermittent preventive treatment
IPTp: intermittent preventive treatment in pregnancy
IRS: insecticide residual spraying
ITN: insecticide treated net
MIS: Malaria Indicator Survey
