# Supplementary material for: State of inequality in malaria intervention coverage in sub-Saharan African countries
Source: BMC Med. 2017 Oct 18;15:185. doi: 10.1186/s12916-017-0948-8 (PMC5646111; doi:10.1186/s12916-017-0948-8)
Supplement: Supplementary file 1 — and SA2 that list by country: country name, ISO3 code, mean population weighted PfPR 2-10, and corresponding DHS/MIS survey used in 2015 tabulations and 2005–2015 year trend analyses. (DOCX 37 kb) [file 12916_2017_948_MOESM1_ESM.docx]

**Additional file 1**

**Table SA1 Country name, ISO3 label, *PfPR_2-10_*, and DHS/MIS survey corresponding to 2015 year label by country**

XX refers to DHS/MIS recode files as follows: KR files were used to estimate fever prevalence, health seeking and treatment for fever among children under the age of 5; PR files were used to estimate proportion of population that slept under an ITN the night prior to the survey and malaria prevalence among children under the age of 5; IR files were used to estimate SP uptake during the most recent pregnancy; HR files were used for estimates of ITN and IRS coverage; WI files were used to obtain asset-wealth index score data for surveys collected prior to 2002. * Country mean malaria parasite prevalence among children aged between 2 and 10 (PfPR2-10) is based on 2015 MAP estimates from (16).

*MAP* Malaria Atlas Project, *DHS* Demographic and Health Survey, *MIS* Malaria Indicator Survey

| Country | ISO3 | *Pf*PR_2-10_* | Survey year(s) | DHS file | Year label |
| --- | --- | --- | --- | --- | --- |
| Angola | AGO | 0.066419 | 2011 | AOXX62FL | 2015 |
| Benin | BEN | 0.226732 | 2011-2012 | BJXX61FL | 2015 |
| Burkina Faso | BFA | 0.339542 | 2014 | BFXX70FL | 2015 |
| Burundi | BDI | 0.076145 | 2012-2013 | BUXX6HFL | 2015 |
| Cameroon | CMR | 0.1459 | 2011 | CMXX61FL | 2015 |
| Chad | TCD | 0.062353 | 2014-2015 | TDXX71FL | 2015 |
| Comoros | COM | 0.164316 | 2012 | KMXX61FL | 2015 |
| Congo | COG | 0.11469 | 2011-2012 | CGXX60FL | 2015 |
| Congo, Democratic Republic | COD | 0.209264 | 2013-2014 | CDXX61FL | 2015 |
| Cote d'Ivoire | CIV | 0.381616 | 2011-2012 | CIXX62FL | 2015 |
| Gabon | GAB | 0.151068 | 2012 | GAXX60FL | 2015 |
| Ghana | GHA | 0.250699 | 2014 | GHXX72FL | 2015 |
| Guinea | GIN | 0.421519 | 2012 | GNXX62FL | 2015 |
| Kenya | KEN | 0.0689 | 2015 | KEXX7HFL | 2015 |
| Liberia | LBR | 0.292958 | 2013 | LBXX6AFL | 2015 |
| Madagascar | MDG | 0.032263 | 2016 | MDXX71FL | 2015 |
| Malawi | MWI | 0.121212 | 2015-2016 | MWXX7HFL | 2015 |
| Mali | MLI | 0.454885 | 2012-2013 | MLXX6HFL | 2015 |
| Mozambique | MOZ | 0.231735 | 2011 | MZXX62FL | 2015 |
| Namibia | NAM | 0.030788 | 2013 | NMXX61FL | 2015 |
| Niger | NER | 0.15034 | 2012 | NIXX61FL | 2015 |
| Nigeria | NGA | 0.259483 | 2013 | NGXX6AFL | 2015 |
| Rwanda | RWA | 0.025569 | 2014-2015 | RWXX70FL | 2015 |
| Senegal | SEN | 0.024716 | 2014 | SNXX70FL | 2015 |
| Sierra Leone | SLE | 0.398516 | 2013 | SLXX61FL | 2015 |
| Tanzania | TZA | 0.050974 | 2015-2016 | TZXX7HFL | 2015 |
| Togo | TGO | 0.367213 | 2013-2014 | TGXX61FL | 2015 |
| Uganda | UGA | 0.12953 | 2014-2015 | UGXX72FL | 2015 |
| Zambia | ZMB | 0.10741 | 2013-2014 | ZMXX61FL | 2015 |
| Zimbabwe | ZWE | 0.024222 | 2015 | ZWXX70FL | 2015 |

**Table SA2 Country name, ISO3 label, and DHS/MIS survey corresponding to 2005, 2010 and 2015 year labels by country**

XX refers to DHS/MIS recode files as follows: KR files were used to estimate fever prevalence, health seeking and treatment for fever among children under the age of 5; PR files were used to estimate proportion of population that slept under an ITN the night prior to the survey and malaria prevalence among children under the age of 5; IR files were used to estimate SP uptake during the most recent pregnancy; HR files were used for estimates of ITN and IRS coverage; WI files were used to obtain asset-wealth index score data for surveys collected prior to 2002.

*DHS* Demographic and Health Survey, *MIS* Malaria Indicator Survey

| Country | ISO3 | Survey year(s) | DHS file | Year label |
| --- | --- | --- | --- | --- |
| Angola | AGO | 2006 | AOXX51FL | 2005 |
| Angola | AGO | 2011 | AOXX62FL | 2010 |
| Benin | BEN | 2006 | BJXX51FL | 2005 |
| Benin | BEN | 2011-2012 | BJXX61FL | 2010 |
| Burkina Faso | BFA | 2003 | BFXX43FL | 2005 |
| Burkina Faso | BFA | 2010 | BFXX62FL | 2010 |
| Burkina Faso | BFA | 2014 | BFXX70FL | 2015 |
| Cameroon | CMR | 2004 | CMXX44FL | 2005 |
| Cameroon | CMR | 2011 | CMXX61FL | 2010 |
| Chad | TCD | 2004 | TDXX41FL | 2005 |
| Chad | TCD | 2014-2015 | TDXX71FL | 2015 |
| Congo | COG | 2005 | CGXX51FL | 2005 |
| Congo | COG | 2011-2012 | CGXX60FL | 2010 |
| Democratic Republic of the Congo | COD | 2007 | CDXX50FL | 2010 |
| Democratic Republic of the Congo | COD | 2013-2014 | CDXX61FL | 2015 |
| Cote d'Ivoire | CIV | 2005 | CIXX50FL | 2005 |
| Cote d'Ivoire | CIV | 2011-2012 | CIXX62FL | 2010 |
| Ghana | GHA | 2003 | GHXX4BFL | 2005 |
| Ghana | GHA | 2008 | GHXX5AFL | 2010 |
| Ghana | GHA | 2014 | GHXX72FL | 2015 |
| Guinea | GIN | 2005 | GNXX52FL | 2005 |
| Guinea | GIN | 2012 | GNXX62FL | 2010 |
| Kenya | KEN | 2003 | KEXX42FL | 2005 |
| Kenya | KEN | 2008-2009 | KEXX52FL | 2010 |
| Kenya | KEN | 2015 | KEXX7HFL | 2015 |
| Liberia | LBR | 2006-2007 | LBXX51FL | 2005 |
| Liberia | LBR | 2011 | LBXX61FL | 2010 |
| Liberia | LBR | 2013 | LBXX6AFL | 2015 |
| Madagascar | MDG | 2003-2004 | MDXX41FL | 2005 |
| Madagascar | MDG | 2011 | MDXX61FL | 2010 |
| Madagascar | MDG | 2016 | MDXX71FL | 2015 |
| Malawi | MWI | 2004 | MWXX4DFL | 2005 |
| Malawi | MWI | 2010 | MWXX61FL | 2010 |
| Malawi | MWI | 2015-2016 | MWXX7HFL | 2015 |
| Mali | MLI | 2006 | MLXX53FL | 2005 |
| Mali | MLI | 2012-2013 | MLXX6HFL | 2010 |
| Mozambique | MOZ | 2003-2004 | MZXX41FL | 2005 |
| Mozambique | MOZ | 2011 | MZXX62FL | 2010 |
| Namibia | NAM | 2006-2007 | NMXX51FL | 2010 |
| Namibia | NAM | 2013 | NMXX61FL | 2015 |
| Niger | NER | 2006 | NIXX51FL | 2005 |
| Niger | NER | 2012 | NIXX61FL | 2010 |
| Nigeria | NGA | 2003 | NGXX4BFL | 2005 |
| Nigeria | NGA | 2008 | NGXX53FL | 2010 |
| Nigeria | NGA | 2013 | NGXX6AFL | 2015 |
| Rwanda | RWA | 2005 | RWXX53FL | 2005 |
| Rwanda | RWA | 2010-2011 | RWXX61FL | 2010 |
| Rwanda | RWA | 2014-2015 | RWXX70FL | 2015 |
| Senegal | SEN | 2005 | SNXX4HFL | 2005 |
| Senegal | SEN | 2010-2011 | SNXX61FL | 2010 |
| Senegal | SEN | 2014 | SNXX70FL | 2015 |
| Sierra Leone | SLE | 2008 | SLXX51FL | 2010 |
| Sierra Leone | SLE | 2013 | SLXX61FL | 2015 |
| Tanzania | TZA | 2004-2005 | TZXX4IFL | 2005 |
| Tanzania | TZA | 2009-2010 | TZXX63FL | 2010 |
| Tanzania | TZA | 2015-2016 | TZXX7HFL | 2015 |
| Uganda | UGA | 2006 | UGXX52FL | 2005 |
| Uganda | UGA | 2011 | UGXX60FL | 2010 |
| Uganda | UGA | 2014-2015 | UGXX72FL | 2015 |
| Zambia | ZMB | 2007 | ZMXX51FL | 2010 |
| Zambia | ZMB | 2013-2014 | ZMXX61FL | 2015 |
| Zimbabwe | ZWE | 2005-2006 | ZWXX52FL | 2005 |
| Zimbabwe | ZWE | 2010-2011 | ZWXX62FL | 2010 |
| Zimbabwe | ZWE | 2015 | ZWXX70FL | 2015 |
