# Supplementary material for: State of inequality in malaria intervention coverage in sub-Saharan African countries
Source: BMC Med. 2017 Oct 18;15:185. doi: 10.1186/s12916-017-0948-8 (PMC5646111; doi:10.1186/s12916-017-0948-8)
Supplement: Supplementary file 2 — Tables detailing country estimates corresponding to level and degree of inequality for each of the malaria intervention coverage indicators including ACTs (Tables SA3-SA9). (DOCX 93 kb) [file 12916_2017_948_MOESM2_ESM.docx]

**Additional file 2**

**Table SA3 Distribution of households with at least one ITN for every two persons in 2015***

| **Country** | **Total** | **Q1** | **Q5** | **Difference Q5-Q1** | **Ratio Q5:Q1** | **CIX** | **SII** |
| --- | --- | --- | --- | --- | --- | --- | --- |
| Angola | 0.064 (0.055 to 0.072) | 0.030 (0.020 to 0.040) | 0.079 (0.062 to 0.095) | 0.049 (0.029 to 0.068) | 2.629 (1.601 to 3.657) | 0.049 (0.035 to 0.064) | 0.065 (0.041 to 0.089) |
| Benin | 0.446 (0.434 to 0.458) | 0.425 (0.404 to 0.446) | 0.516 (0.491 to 0.541) | 0.091 (0.059 to 0.124) | 1.215 (1.132 to 1.298) | 0.071 (0.045 to 0.097) | 0.221 (0.152 to 0.290) |
| Burkina Faso | 0.492 (0.476 to 0.507) | 0.411 (0.371 to 0.450) | 0.583 (0.552 to 0.614) | 0.172 (0.122 to 0.223) | 1.419 (1.262 to 1.576) | 0.142 (0.104 to 0.181) | 0.354 (0.261 to 0.446) |
| Burundi | 0.255 (0.230 to 0.280) | 0.207 (0.172 to 0.242) | 0.309 (0.273 to 0.345) | 0.102 (0.055 to 0.148) | 1.492 (1.206 to 1.778) | 0.076 (0.041 to 0.111) | 0.546 (0.367 to 0.725) |
| Cameroon | 0.045 (0.041 to 0.049) | 0.036 (0.024 to 0.048) | 0.063 (0.054 to 0.071) | 0.026 (0.012 to 0.041) | 1.730 (1.100 to 2.359) | 0.022 (0.012 to 0.033) | 0.060 (0.031 to 0.089) |
| Chad | 0.280 (0.270 to 0.291) | 0.295 (0.274 to 0.316) | 0.350 (0.327 to 0.372) | 0.055 (0.023 to 0.086) | 1.186 (1.070 to 1.302) | 0.019 (-0.005 to 0.043) | 0.231 (0.159 to 0.303) |
| Comoros | 0.254 (0.230 to 0.277) | 0.186 (0.154 to 0.219) | 0.321 (0.277 to 0.364) | 0.134 (0.081 to 0.187) | 1.719 (1.352 to 2.086) | 0.117 (0.077 to 0.157) | 0.281 (0.193 to 0.370) |
| Congo | 0.111 (0.100 to 0.122) | 0.174 (0.152 to 0.196) | 0.084 (0.066 to 0.103) | -0.090 (-0.118 to -0.061) | 0.485 (0.362 to 0.609) | -0.069 (-0.091 to -0.048) | -0.119 (-0.153 to -0.084) |
| Congo, Democratic Republic | 0.254 (0.238 to 0.269) | 0.233 (0.207 to 0.260) | 0.237 (0.208 to 0.267) | 0.004 (-0.035 to 0.044) | 1.018 (0.847 to 1.189) | 0.020 (-0.012 to 0.051) | -0.012 (-0.085 to 0.062) |
| Cote d’Ivoire | 0.317 (0.299 to 0.335) | 0.345 (0.304 to 0.385) | 0.280 (0.242 to 0.319) | -0.064 (-0.120 to -0.008) | 0.814 (0.666 to 0.962) | -0.045 (-0.088 to -0.003) | -0.135 (-0.236 to -0.034) |
| Gabon | 0.145 (0.134 to 0.156) | 0.213 (0.193 to 0.232) | 0.064 (0.046 to 0.083) | -0.148 (-0.175 to -0.121) | 0.303 (0.210 to 0.396) | -0.112 (-0.134 to -0.089) | -0.231 (-0.273 to -0.189) |
| Ghana | 0.452 (0.436 to 0.469) | 0.426 (0.396 to 0.456) | 0.411 (0.375 to 0.447) | -0.015 (-0.062 to 0.032) | 0.965 (0.857 to 1.074) | -0.052 (-0.089 to -0.016) | -0.130 (-0.250 to -0.009) |
| Guinea | 0.097 (0.087 to 0.107) | 0.085 (0.067 to 0.102) | 0.076 (0.060 to 0.093) | -0.008 (-0.033 to 0.016) | 0.901 (0.629 to 1.173) | 0.004 (-0.016 to 0.024) | -0.008 (-0.054 to 0.037) |
| Kenya | 0.400 (0.367 to 0.434) | 0.258 (0.214 to 0.303) | 0.531 (0.470 to 0.592) | 0.272 (0.196 to 0.349) | 2.054 (1.624 to 2.484) | 0.213 (0.153 to 0.273) | 0.550 (0.426 to 0.674) |
| Liberia | 0.221 (0.204 to 0.239) | 0.227 (0.198 to 0.257) | 0.187 (0.157 to 0.217) | -0.040 (-0.082 to 0.002) | 0.823 (0.653 to 0.993) | -0.052 (-0.088 to -0.015) | -0.121 (-0.197 to -0.045) |
| Madagascar | 0.444 (0.420 to 0.468) | 0.422 (0.386 to 0.458) | 0.456 (0.408 to 0.504) | 0.034 (-0.026 to 0.094) | 1.082 (0.935 to 1.228) | 0.032 (-0.013 to 0.078) | 0.086 (-0.064 to 0.236) |
| Malawi | 0.235 (0.224 to 0.245) | 0.153 (0.139 to 0.167) | 0.383 (0.363 to 0.402) | 0.230 (0.206 to 0.254) | 2.502 (2.241 to 2.763) | 0.170 (0.153 to 0.186) | 0.481 (0.424 to 0.538) |
| Mali | 0.418 (0.402 to 0.435) | 0.370 (0.336 to 0.405) | 0.438 (0.407 to 0.469) | 0.068 (0.023 to 0.113) | 1.183 (1.048 to 1.318) | 0.046 (0.012 to 0.081) | 0.106 (0.002 to 0.210) |
| Mozambique | 0.226 (0.211 to 0.241) | 0.169 (0.143 to 0.196) | 0.278 (0.252 to 0.303) | 0.108 (0.071 to 0.146) | 1.639 (1.337 to 1.941) | 0.090 (0.061 to 0.119) | 0.168 (0.109 to 0.228) |
| Namibia | 0.120 (0.110 to 0.130) | 0.149 (0.123 to 0.175) | 0.068 (0.048 to 0.088) | -0.081 (-0.114 to -0.048) | 0.458 (0.301 to 0.615) | -0.068 (-0.093 to -0.044) | -0.123 (-0.163 to -0.084) |
| Niger | 0.166 (0.156 to 0.177) | 0.107 (0.091 to 0.123) | 0.262 (0.241 to 0.283) | 0.155 (0.129 to 0.181) | 2.451 (2.041 to 2.861) | 0.124 (0.105 to 0.144) | 0.545 (0.458 to 0.633) |
| Nigeria | 0.221 (0.210 to 0.232) | 0.208 (0.181 to 0.236) | 0.199 (0.183 to 0.216) | -0.009 (-0.041 to 0.023) | 0.956 (0.807 to 1.104) | -0.017 (-0.041 to 0.007) | -0.045 (-0.098 to 0.009) |
| Rwanda | 0.426 (0.409 to 0.442) | 0.307 (0.284 to 0.329) | 0.601 (0.571 to 0.630) | 0.294 (0.258 to 0.330) | 1.959 (1.791 to 2.127) | 0.227 (0.201 to 0.254) | 0.600 (0.544 to 0.656) |
| Senegal | 0.363 (0.331 to 0.395) | 0.338 (0.295 to 0.381) | 0.308 (0.234 to 0.382) | -0.030 (-0.116 to 0.056) | 0.911 (0.661 to 1.161) | -0.047 (-0.119 to 0.025) | -0.080 (-0.214 to 0.054) |
| Sierra Leone | 0.149 (0.140 to 0.159) | 0.155 (0.136 to 0.173) | 0.157 (0.136 to 0.179) | 0.003 (-0.025 to 0.031) | 1.018 (0.833 to 1.202) | 0.008 (-0.013 to 0.030) | 0.081 (-0.002 to 0.164) |
| Tanzania | 0.388 (0.373 to 0.402) | 0.279 (0.247 to 0.311) | 0.428 (0.400 to 0.457) | 0.149 (0.106 to 0.193) | 1.536 (1.329 to 1.743) | 0.102 (0.068 to 0.136) | 0.219 (0.118 to 0.319) |
| Togo | 0.329 (0.313 to 0.346) | 0.272 (0.245 to 0.299) | 0.325 (0.293 to 0.358) | 0.054 (0.012 to 0.096) | 1.197 (1.029 to 1.365) | 0.009 (-0.024 to 0.043) | 0.016 (-0.085 to 0.117) |
| Uganda | 0.623 (0.600 to 0.645) | 0.618 (0.575 to 0.662) | 0.622 (0.580 to 0.664) | 0.004 (-0.058 to 0.065) | 1.006 (0.905 to 1.106) | 0.001 (-0.050 to 0.051) | 0.018 (-0.103 to 0.138) |
| Zambia | 0.274 (0.262 to 0.285) | 0.248 (0.228 to 0.267) | 0.329 (0.298 to 0.360) | 0.082 (0.045 to 0.118) | 1.329 (1.166 to 1.493) | 0.061 (0.033 to 0.088) | 0.225 (0.132 to 0.317) |
| Zimbabwe | 0.264 (0.244 to 0.284) | 0.268 (0.234 to 0.303) | 0.204 (0.172 to 0.235) | -0.065 (-0.111 to -0.020) | 0.758 (0.610 to 0.906) | -0.068 (-0.104 to -0.032) | -0.176 (-0.250 to -0.103) |

For each country population weighted and adjusted for survey design estimate of the statistic characterizing the level and distribution of the respective malaria intervention coverage indictor is reported in each column. 95% confidence intervals are reported in the parentheses below the estimate. Q1 and Q5 denote respectively the lowest and highest asset-wealth quintiles. CIX was implemented with *conindex* command in Stata SE 14. SII was computed on individual data; estimates represent the difference in the predicted probabilities of the respective coverage indicator evaluated at highest and lowest values of the asset-wealth ranking variable (1 and 0) computed as marginal effects following probit estimation. For details of statistics evaluated refer to text and methodological guidance in [29]. *Data drawn from a subset of countries with DHS/MIS conducted after 2010 (country list and year of data collection are detailed in Additional file 1).

*CIX* Concentration Index, *SII* Slope Index of Inequality

**Table SA4 Distribution of population that slept under an ITN last night in 2015***

| **Country** | **Total** | **Q1** | **Q5** | **Difference Q5-Q1** | **Ratio Q5:Q1** | **CIX** | **SII** |
| --- | --- | --- | --- | --- | --- | --- | --- |
| Angola | 0.189 (0.172 to 0.206) | 0.100 (0.077 to 0.122) | 0.173 (0.153 to 0.194) | 0.073 (0.043 to 0.104) | 1.737 (1.298 to 2.177) | 0.073 (0.045 to 0.102) | 0.092 (0.049 to 0.134) |
| Benin | 0.627 (0.616 to 0.638) | 0.613 (0.591 to 0.635) | 0.655 (0.635 to 0.676) | 0.042 (0.012 to 0.072) | 1.068 (1.017 to 1.119) | 0.021 (-0.002 to 0.044) | 0.054 (-0.010 to 0.118) |
| Burkina Faso | 0.670 (0.655 to 0.685) | 0.637 (0.606 to 0.668) | 0.610 (0.578 to 0.643) | -0.026 (-0.072 to 0.019) | 0.958 (0.889 to 1.028) | -0.014 (-0.050 to 0.023) | -0.159 (-0.271 to -0.048) |
| Burundi | 0.486 (0.451 to 0.521) | 0.351 (0.306 to 0.395) | 0.575 (0.528 to 0.622) | 0.224 (0.170 to 0.279) | 1.640 (1.426 to 1.854) | 0.168 (0.125 to 0.211) | 0.596 (0.475 to 0.717) |
| Cameroon | 0.076 (0.071 to 0.082) | 0.045 (0.036 to 0.054) | 0.099 (0.087 to 0.111) | 0.054 (0.038 to 0.069) | 2.178 (1.670 to 2.685) | 0.040 (0.028 to 0.051) | 0.086 (0.056 to 0.117) |
| Chad | 0.217 (0.204 to 0.230) | 0.208 (0.184 to 0.233) | 0.318 (0.292 to 0.343) | 0.110 (0.075 to 0.145) | 1.527 (1.311 to 1.743) | 0.059 (0.030 to 0.088) | 0.381 (0.306 to 0.456) |
| Comoros | 0.384 (0.363 to 0.405) | 0.339 (0.302 to 0.377) | 0.396 (0.353 to 0.438) | 0.056 (0.000 to 0.112) | 1.165 (0.989 to 1.341) | 0.058 (0.016 to 0.100) | 0.145 (0.044 to 0.246) |
| Congo | 0.261 (0.244 to 0.277) | 0.370 (0.339 to 0.401) | 0.174 (0.146 to 0.203) | -0.196 (-0.237 to -0.154) | 0.471 (0.385 to 0.557) | -0.157 (-0.189 to -0.125) | -0.275 (-0.326 to -0.224) |
| Congo, Democratic Republic | 0.502 (0.480 to 0.524) | 0.421 (0.384 to 0.458) | 0.455 (0.415 to 0.495) | 0.034 (-0.021 to 0.088) | 1.080 (0.947 to 1.214) | 0.035 (-0.010 to 0.080) | -0.038 (-0.132 to 0.056) |
| Cote d’Ivoire | 0.333 (0.313 to 0.352) | 0.426 (0.383 to 0.469) | 0.218 (0.185 to 0.252) | -0.208 (-0.262 to -0.154) | 0.512 (0.418 to 0.606) | -0.169 (-0.214 to -0.124) | -0.375 (-0.448 to -0.303) |
| Gabon | 0.267 (0.250 to 0.284) | 0.318 (0.296 to 0.339) | 0.116 (0.092 to 0.139) | -0.202 (-0.235 to -0.169) | 0.364 (0.284 to 0.444) | -0.167 (-0.198 to -0.136) | -0.324 (-0.384 to -0.265) |
| Ghana | 0.357 (0.339 to 0.374) | 0.463 (0.425 to 0.501) | 0.181 (0.159 to 0.202) | -0.282 (-0.326 to -0.239) | 0.390 (0.334 to 0.447) | -0.267 (-0.303 to -0.231) | -0.703 (-0.777 to -0.629) |
| Guinea | 0.189 (0.176 to 0.202) | 0.190 (0.160 to 0.220) | 0.141 (0.119 to 0.162) | -0.049 (-0.086 to -0.012) | 0.741 (0.579 to 0.904) | -0.030 (-0.058 to -0.002) | -0.094 (-0.158 to -0.031) |
| Kenya | 0.476 (0.442 to 0.510) | 0.345 (0.290 to 0.400) | 0.539 (0.463 to 0.615) | 0.194 (0.099 to 0.288) | 1.562 (1.226 to 1.897) | 0.140 (0.066 to 0.213) | 0.381 (0.209 to 0.553) |
| Liberia | 0.317 (0.295 to 0.340) | 0.311 (0.279 to 0.342) | 0.217 (0.175 to 0.258) | -0.094 (-0.146 to -0.042) | 0.697 (0.546 to 0.848) | -0.094 (-0.141 to -0.046) | -0.232 (-0.330 to -0.134) |
| Madagascar | 0.682 (0.658 to 0.706) | 0.791 (0.767 to 0.814) | 0.604 (0.553 to 0.655) | -0.187 (-0.244 to -0.129) | 0.764 (0.694 to 0.834) | -0.151 (-0.195 to -0.108) | -0.436 (-0.581 to -0.291) |
| Malawi | 0.339 (0.327 to 0.351) | 0.249 (0.230 to 0.268) | 0.451 (0.428 to 0.475) | 0.203 (0.174 to 0.231) | 1.815 (1.654 to 1.975) | 0.149 (0.128 to 0.170) | 0.355 (0.285 to 0.425) |
| Mali | 0.605 (0.591 to 0.620) | 0.563 (0.533 to 0.593) | 0.590 (0.563 to 0.617) | 0.027 (-0.013 to 0.068) | 1.049 (0.975 to 1.122) | 0.024 (-0.008 to 0.055) | -0.053 (-0.148 to 0.043) |
| Mozambique | 0.295 (0.280 to 0.309) | 0.262 (0.230 to 0.294) | 0.335 (0.307 to 0.363) | 0.073 (0.030 to 0.116) | 1.278 (1.087 to 1.469) | 0.056 (0.023 to 0.090) | 0.112 (0.050 to 0.175) |
| Namibia | 0.039 (0.034 to 0.045) | 0.060 (0.043 to 0.076) | 0.014 (0.008 to 0.020) | -0.046 (-0.063 to -0.029) | 0.232 (0.112 to 0.352) | -0.039 (-0.051 to -0.026) | -0.064 (-0.081 to -0.047) |
| Niger | 0.138 (0.125 to 0.151) | 0.064 (0.052 to 0.077) | 0.239 (0.214 to 0.263) | 0.174 (0.147 to 0.201) | 3.702 (2.908 to 4.496) | 0.144 (0.123 to 0.164) | 0.533 (0.426 to 0.640) |
| Nigeria | 0.129 (0.120 to 0.139) | 0.094 (0.076 to 0.113) | 0.120 (0.109 to 0.131) | 0.025 (0.004 to 0.047) | 1.267 (0.995 to 1.539) | 0.011 (-0.008 to 0.031) | 0.014 (-0.029 to 0.058) |
| Rwanda | 0.614 (0.597 to 0.631) | 0.472 (0.445 to 0.500) | 0.736 (0.710 to 0.762) | 0.263 (0.227 to 0.299) | 1.557 (1.456 to 1.658) | 0.218 (0.191 to 0.244) | 0.457 (0.397 to 0.517) |
| Senegal | 0.404 (0.367 to 0.441) | 0.368 (0.304 to 0.432) | 0.317 (0.226 to 0.409) | -0.051 (-0.163 to 0.061) | 0.862 (0.570 to 1.154) | -0.047 (-0.131 to 0.037) | -0.103 (-0.266 to 0.059) |
| Sierra Leone | 0.418 (0.400 to 0.436) | 0.440 (0.412 to 0.469) | 0.286 (0.258 to 0.314) | -0.154 (-0.195 to -0.114) | 0.649 (0.573 to 0.725) | -0.103 (-0.136 to -0.071) | -0.410 (-0.477 to -0.342) |
| Tanzania | 0.490 (0.475 to 0.506) | 0.441 (0.402 to 0.481) | 0.528 (0.495 to 0.561) | 0.087 (0.034 to 0.139) | 1.196 (1.064 to 1.329) | 0.069 (0.029 to 0.110) | 0.158 (0.046 to 0.269) |
| Togo | 0.336 (0.321 to 0.352) | 0.360 (0.330 to 0.389) | 0.280 (0.256 to 0.305) | -0.080 (-0.118 to -0.041) | 0.778 (0.685 to 0.872) | -0.082 (-0.113 to -0.051) | -0.207 (-0.284 to -0.131) |
| Uganda | 0.686 (0.666 to 0.705) | 0.723 (0.688 to 0.759) | 0.638 (0.596 to 0.680) | -0.085 (-0.142 to -0.029) | 0.882 (0.807 to 0.957) | -0.085 (-0.128 to -0.042) | -0.190 (-0.305 to -0.075) |
| Zambia | 0.349 (0.336 to 0.362) | 0.347 (0.325 to 0.369) | 0.344 (0.315 to 0.373) | -0.003 (-0.040 to 0.033) | 0.990 (0.885 to 1.095) | -0.022 (-0.051 to 0.006) | -0.054 (-0.136 to 0.029) |
| Zimbabwe | 0.085 (0.075 to 0.096) | 0.098 (0.078 to 0.117) | 0.066 (0.052 to 0.079) | -0.032 (-0.056 to -0.008) | 0.672 (0.477 to 0.867) | -0.034 (-0.054 to -0.014) | -0.072 (-0.109 to -0.034) |

For each country population weighted and adjusted for survey design estimate of the statistic characterizing the level and distribution of the respective malaria intervention coverage indictor is reported in each column. 95% confidence intervals are reported in the parentheses below the estimate. Q1 and Q5 denote respectively the lowest and highest asset-wealth quintiles. CIX was implemented with *conindex* command in Stata SE 14. SII was computed on individual data; estimates represent the difference in the predicted probabilities of the respective coverage indicator evaluated at highest and lowest values of the asset-wealth ranking variable (1 and 0) computed as marginal effects following probit estimation. For details of statistics evaluated refer to text and methodological guidance in [29]. *Data drawn from a subset of countries with DHS/MIS conducted after 2010 (country list and year of data collection are detailed in Additional file 1).

*CIX* Concentration Index, *SII* Slope Index of Inequality

**Table SA5 Distribution of households with IRS in the last 12 months in 2015***

| **Country** | **Total** | **Q1** | **Q5** | **Difference Q5-Q1** | **Ratio Q5:Q1** | **CIX** | **SII** |
| --- | --- | --- | --- | --- | --- | --- | --- |
| Angola |  |  |  |  |  |  |  |
| Benin | 0.060 (0.052 to 0.069) | 0.124 (0.100 to 0.148) | 0.030 (0.019 to 0.041) | -0.094 (-0.121 to -0.068) | 0.241 (0.142 to 0.340) | -0.074 (-0.094 to -0.053) | -0.186 (-0.253 to -0.118) |
| Burkina Faso | 0.004 (0.002 to 0.006) | 0.000 (0.000 to 0.000) | 0.009 (0.003 to 0.016) | 0.009 (0.003 to 0.016) |  | 0.007 (0.002 to 0.013) | 0.042 (-0.009 to 0.093) |
| Burundi | 0.053 (0.025 to 0.081) | 0.082 (0.033 to 0.132) | 0.016 (0.005 to 0.026) | -0.067 (-0.115 to -0.018) | 0.189 (0.035 to 0.343) | -0.052 (-0.090 to -0.014) | -0.286 (-0.558 to -0.014) |
| Cameroon | 0.013 (0.010 to 0.016) | 0.002 (0.000 to 0.004) | 0.033 (0.025 to 0.042) | 0.031 (0.022 to 0.040) | 16.464 (1.072 to 31.856) | 0.028 (0.022 to 0.035) | 0.133 (0.091 to 0.175) |
| Chad | 0.004 (0.003 to 0.006) | 0.001 (0.000 to 0.003) | 0.010 (0.007 to 0.014) | 0.009 (0.005 to 0.013) | 7.687 (-0.234 to 15.609) | 0.007 (0.004 to 0.010) | 0.053 (0.020 to 0.086) |
| Comoros | 0.043 (0.037 to 0.049) | 0.094 (0.073 to 0.114) | 0.018 (0.010 to 0.026) | -0.075 (-0.099 to -0.051) | 0.197 (0.094 to 0.300) | -0.063 (-0.080 to -0.046) | -0.186 (-0.248 to -0.124) |
| Congo |  |  |  |  |  |  |  |
| Congo, Democratic Republic |  |  |  |  |  |  |  |
| Cote d’Ivoire | 0.015 (0.006 to 0.023) | 0.009 (-0.005 to 0.023) | 0.030 (0.016 to 0.044) | 0.021 (0.004 to 0.039) | 3.454 (-2.037 to 8.946) | 0.018 (0.007 to 0.030) | 0.081 (0.002 to 0.160) |
| Gabon | 0.042 (0.033 to 0.052) | 0.005 (0.002 to 0.007) | 0.100 (0.071 to 0.129) | 0.095 (0.066 to 0.124) | 20.826 (8.650 to 33.002) | 0.072 (0.051 to 0.094) | 0.191 (0.130 to 0.252) |
| Ghana | 0.097 (0.073 to 0.121) | 0.292 (0.216 to 0.369) | 0.055 (0.031 to 0.079) | -0.237 (-0.317 to -0.157) | 0.188 (0.093 to 0.283) | -0.134 (-0.187 to -0.081) | -0.628 (-0.849 to -0.406) |
| Guinea | 0.017 (0.005 to 0.030) | 0.005 (-0.001 to 0.012) | 0.036 (0.020 to 0.052) | 0.030 (0.013 to 0.048) | 6.482 (-1.828 to 14.792) | 0.025 (0.012 to 0.037) | 0.060 (0.013 to 0.108) |
| Kenya |  |  |  |  |  |  |  |
| Liberia | 0.107 (0.076 to 0.137) | 0.169 (0.112 to 0.225) | 0.049 (0.029 to 0.069) | -0.120 (-0.180 to -0.060) | 0.289 (0.134 to 0.444) | -0.102 (-0.157 to -0.048) | -0.182 (-0.268 to -0.095) |
| Madagascar | 0.087 (0.065 to 0.109) | 0.086 (0.052 to 0.121) | 0.061 (0.040 to 0.082) | -0.025 (-0.065 to 0.014) | 0.704 (0.341 to 1.068) | -0.023 (-0.058 to 0.012) | -0.113 (-0.207 to -0.019) |
| Malawi | 0.049 (0.039 to 0.060) | 0.045 (0.033 to 0.057) | 0.052 (0.037 to 0.067) | 0.007 (-0.010 to 0.025) | 1.161 (0.747 to 1.575) | 0.002 (-0.012 to 0.015) | 0.005 (-0.034 to 0.044) |
| Mali | 0.062 (0.046 to 0.078) | 0.058 (0.037 to 0.079) | 0.057 (0.042 to 0.071) | -0.002 (-0.027 to 0.024) | 0.975 (0.548 to 1.401) | -0.003 (-0.025 to 0.018) | 0.041 (-0.058 to 0.139) |
| Mozambique | 0.185 (0.164 to 0.205) | 0.143 (0.099 to 0.187) | 0.301 (0.276 to 0.326) | 0.158 (0.108 to 0.208) | 2.105 (1.434 to 2.775) | 0.133 (0.092 to 0.174) | 0.321 (0.242 to 0.400) |
| Namibia | 0.155 (0.141 to 0.169) | 0.313 (0.277 to 0.348) | 0.030 (0.021 to 0.038) | -0.283 (-0.320 to -0.246) | 0.094 (0.064 to 0.125) | -0.224 (-0.254 to -0.193) | -0.331 (-0.372 to -0.289) |
| Niger | 0.005 (0.003 to 0.006) | 0.001 (-0.001 to 0.003) | 0.015 (0.010 to 0.021) | 0.014 (0.008 to 0.020) | 11.421 (-5.643 to 28.484) | 0.010 (0.006 to 0.014) | 0.233 (0.130 to 0.337) |
| Nigeria | 0.017 (0.011 to 0.023) | 0.012 (-0.000 to 0.024) | 0.021 (0.014 to 0.028) | 0.009 (-0.005 to 0.023) | 1.767 (-0.125 to 3.658) | 0.010 (-0.001 to 0.022) | 0.027 (-0.001 to 0.056) |
| Rwanda |  |  |  |  |  |  |  |
| Senegal | 0.087 (0.059 to 0.115) | 0.150 (0.091 to 0.209) | 0.071 (0.042 to 0.100) | -0.079 (-0.143 to -0.014) | 0.474 (0.212 to 0.735) | -0.062 (-0.112 to -0.011) | -0.103 (-0.192 to -0.014) |
| Sierra Leone | 0.048 (0.031 to 0.065) | 0.035 (0.015 to 0.054) | 0.069 (0.050 to 0.088) | 0.034 (0.007 to 0.061) | 1.996 (0.751 to 3.240) | 0.024 (0.001 to 0.047) | 0.168 (-0.000 to 0.336) |
| Tanzania | 0.055 (0.046 to 0.064) | 0.039 (0.021 to 0.057) | 0.061 (0.046 to 0.075) | 0.022 (-0.002 to 0.046) | 1.563 (0.728 to 2.399) | 0.009 (-0.011 to 0.029) | 0.025 (-0.031 to 0.081) |
| Togo |  |  |  |  |  |  |  |
| Uganda | 0.049 (0.032 to 0.066) | 0.123 (0.073 to 0.173) | 0.014 (0.004 to 0.023) | -0.110 (-0.162 to -0.057) | 0.109 (0.013 to 0.206) | -0.087 (-0.129 to -0.046) | -0.182 (-0.294 to -0.069) |
| Zambia | 0.284 (0.266 to 0.303) | 0.227 (0.195 to 0.259) | 0.348 (0.308 to 0.387) | 0.120 (0.069 to 0.171) | 1.530 (1.252 to 1.808) | 0.098 (0.059 to 0.138) | 0.285 (0.153 to 0.418) |
| Zimbabwe | 0.213 (0.179 to 0.248) | 0.376 (0.315 to 0.437) | 0.087 (0.030 to 0.143) | -0.290 (-0.372 to -0.208) | 0.230 (0.076 to 0.384) | -0.261 (-0.327 to -0.195) | -0.433 (-0.524 to -0.342) |

For each country population weighted and adjusted for survey design estimate of the statistic characterizing the level and distribution of the respective malaria intervention coverage indictor is reported in each column. 95% confidence intervals are reported in the parentheses below the estimate. Q1 and Q5 denote respectively the lowest and highest asset-wealth quintiles. CIX was implemented with *conindex* command in Stata SE 14. SII was computed on individual data; estimates represent the difference in the predicted probabilities of the respective coverage indicator evaluated at highest and lowest values of the asset-wealth ranking variable (1 and 0) computed as marginal effects following probit estimation. For details of statistics evaluated refer to text and methodological guidance in [29]. *Data drawn from a subset of countries with DHS/MIS conducted after 2010 (country list and year of data collection are detailed in Additional file 1).

*CIX* Concentration Index, *SII* Slope Index of Inequality

**Table SA6 Distribution of women that received at least 3 doses of SP during an ANC visit during their most recent pregnancy in 2015***

| **Country** | **Total** | **Q1** | **Q5** | **Difference Q5-Q1** | **Ratio Q5:Q1** | **CIX** | **SII** |
| --- | --- | --- | --- | --- | --- | --- | --- |
| Angola | 0.079 (0.067 to 0.091) | 0.017 (0.004 to 0.030) | 0.171 (0.126 to 0.215) | 0.154 (0.108 to 0.200) | 10.126 (2.093 to 18.159) | 0.131 (0.104 to 0.158) | 0.231 (0.178 to 0.284) |
| Benin | 0.139 (0.126 to 0.152) | 0.098 (0.078 to 0.118) | 0.207 (0.173 to 0.241) | 0.109 (0.069 to 0.149) | 2.112 (1.556 to 2.668) | 0.083 (0.053 to 0.113) | 0.240 (0.149 to 0.331) |
| Burkina Faso |  |  |  |  |  |  |  |
| Burundi |  |  |  |  |  |  |  |
| Cameroon | 0.115 (0.103 to 0.127) | 0.065 (0.047 to 0.084) | 0.190 (0.156 to 0.224) | 0.124 (0.086 to 0.163) | 2.895 (1.925 to 3.866) | 0.088 (0.062 to 0.114) | 0.239 (0.156 to 0.321) |
| Chad | 0.076 (0.065 to 0.086) | 0.055 (0.040 to 0.070) | 0.151 (0.121 to 0.181) | 0.096 (0.062 to 0.129) | 2.752 (1.820 to 3.684) | 0.055 (0.032 to 0.077) | 0.294 (0.186 to 0.403) |
| Comoros | 0.101 (0.080 to 0.123) | 0.080 (0.040 to 0.120) | 0.114 (0.058 to 0.170) | 0.034 (-0.036 to 0.103) | 1.423 (0.414 to 2.433) | 0.012 (-0.040 to 0.064) | 0.041 (-0.090 to 0.172) |
| Congo | 0.105 (0.089 to 0.120) | 0.089 (0.073 to 0.105) | 0.095 (0.052 to 0.138) | 0.006 (-0.040 to 0.051) | 1.063 (0.549 to 1.577) | 0.006 (-0.023 to 0.035) | 0.004 (-0.056 to 0.065) |
| Congo, Democratic Republic | 0.054 (0.043 to 0.064) | 0.046 (0.029 to 0.063) | 0.059 (0.035 to 0.084) | 0.014 (-0.016 to 0.043) | 1.294 (0.587 to 2.000) | 0.016 (-0.005 to 0.037) | 0.028 (-0.037 to 0.093) |
| Cote d’Ivoire | 0.066 (0.054 to 0.079) | 0.054 (0.035 to 0.072) | 0.100 (0.064 to 0.136) | 0.046 (0.006 to 0.086) | 1.862 (0.942 to 2.783) | 0.032 (0.004 to 0.060) | 0.108 (0.003 to 0.213) |
| Gabon | 0.063 (0.045 to 0.081) | 0.049 (0.034 to 0.065) | 0.066 (0.014 to 0.117) | 0.016 (-0.037 to 0.069) | 1.327 (0.217 to 2.437) | 0.005 (-0.032 to 0.042) | 0.012 (-0.067 to 0.091) |
| Ghana | 0.385 (0.352 to 0.418) | 0.366 (0.294 to 0.438) | 0.507 (0.447 to 0.566) | 0.140 (0.047 to 0.234) | 1.384 (1.067 to 1.701) | 0.080 (0.010 to 0.150) | 0.229 (0.045 to 0.413) |
| Guinea | 0.094 (0.080 to 0.109) | 0.045 (0.028 to 0.062) | 0.150 (0.116 to 0.184) | 0.105 (0.067 to 0.142) | 3.320 (1.863 to 4.777) | 0.090 (0.062 to 0.117) | 0.208 (0.138 to 0.278) |
| Kenya | 0.240 (0.203 to 0.277) | 0.259 (0.190 to 0.327) | 0.261 (0.154 to 0.367) | 0.002 (-0.123 to 0.128) | 1.009 (0.522 to 1.496) | -0.002 (-0.093 to 0.089) | 0.032 (-0.204 to 0.267) |
| Liberia | 0.172 (0.149 to 0.195) | 0.172 (0.140 to 0.203) | 0.127 (0.083 to 0.172) | -0.044 (-0.099 to 0.010) | 0.741 (0.450 to 1.033) | -0.039 (-0.085 to 0.006) | -0.111 (-0.211 to -0.012) |
| Madagascar | 0.101 (0.085 to 0.117) | 0.087 (0.063 to 0.111) | 0.113 (0.073 to 0.153) | 0.026 (-0.019 to 0.071) | 1.299 (0.740 to 1.858) | 0.032 (-0.000 to 0.063) | 0.071 (-0.074 to 0.216) |
| Malawi | 0.304 (0.289 to 0.319) | 0.306 (0.277 to 0.335) | 0.288 (0.247 to 0.330) | -0.018 (-0.069 to 0.033) | 0.942 (0.779 to 1.106) | 0.008 (-0.028 to 0.045) | -0.003 (-0.100 to 0.094) |
| Mali | 0.108 (0.092 to 0.123) | 0.054 (0.034 to 0.074) | 0.224 (0.183 to 0.265) | 0.170 (0.124 to 0.216) | 4.125 (2.440 to 5.810) | 0.134 (0.101 to 0.167) | 0.592 (0.452 to 0.731) |
| Mozambique | 0.094 (0.082 to 0.107) | 0.072 (0.049 to 0.095) | 0.122 (0.094 to 0.150) | 0.049 (0.013 to 0.086) | 1.686 (1.007 to 2.365) | 0.051 (0.024 to 0.078) | 0.101 (0.036 to 0.166) |
| Namibia | 0.033 (0.023 to 0.042) | 0.035 (0.016 to 0.054) | 0.015 (0.001 to 0.031) | -0.020 (-0.044 to 0.005) | 0.442 (-0.052 to 0.936) | -0.001 (-0.020 to 0.017) | -0.010 (-0.045 to 0.024) |
| Niger | 0.086 (0.073 to 0.099) | 0.059 (0.040 to 0.078) | 0.135 (0.105 to 0.165) | 0.076 (0.041 to 0.111) | 2.280 (1.401 to 3.160) | 0.049 (0.024 to 0.075) | 0.239 (0.081 to 0.397) |
| Nigeria | 0.058 (0.050 to 0.066) | 0.025 (0.017 to 0.032) | 0.063 (0.049 to 0.076) | 0.038 (0.022 to 0.054) | 2.546 (1.604 to 3.489) | 0.040 (0.027 to 0.053) | 0.083 (0.049 to 0.118) |
| Rwanda |  |  |  |  |  |  |  |
| Senegal | 0.034 (0.022 to 0.046) | 0.025 (0.013 to 0.037) | 0.011 (-0.005 to 0.027) | -0.014 (-0.034 to 0.006) | 0.443 (-0.220 to 1.105) | 0.004 (-0.015 to 0.023) | 0.001 (-0.038 to 0.041) |
| Sierra Leone | 0.201 (0.175 to 0.227) | 0.169 (0.138 to 0.201) | 0.153 (0.109 to 0.197) | -0.016 (-0.070 to 0.037) | 0.903 (0.596 to 1.211) | 0.002 (-0.037 to 0.040) | -0.130 (-0.272 to 0.012) |
| Tanzania | 0.078 (0.067 to 0.090) | 0.055 (0.037 to 0.074) | 0.130 (0.101 to 0.159) | 0.074 (0.040 to 0.109) | 2.345 (1.394 to 3.296) | 0.049 (0.023 to 0.076) | 0.191 (0.083 to 0.299) |
| Togo | 0.190 (0.167 to 0.213) | 0.103 (0.070 to 0.135) | 0.321 (0.264 to 0.377) | 0.218 (0.153 to 0.283) | 3.118 (1.985 to 4.250) | 0.183 (0.133 to 0.233) | 0.600 (0.449 to 0.750) |
| Uganda | 0.252 (0.223 to 0.281) | 0.230 (0.182 to 0.279) | 0.268 (0.210 to 0.326) | 0.038 (-0.044 to 0.119) | 1.164 (0.785 to 1.542) | 0.020 (-0.041 to 0.081) | 0.031 (-0.141 to 0.204) |
| Zambia | 0.496 (0.476 to 0.516) | 0.419 (0.385 to 0.454) | 0.643 (0.598 to 0.689) | 0.224 (0.167 to 0.281) | 1.534 (1.368 to 1.699) | 0.166 (0.126 to 0.207) | 0.456 (0.374 to 0.539) |
| Zimbabwe | 0.079 (0.067 to 0.091) | 0.017 (0.004 to 0.030) | 0.171 (0.126 to 0.215) | 0.154 (0.108 to 0.200) | 10.126 (2.093 to 18.159) | 0.131 (0.104 to 0.158) | 0.231 (0.178 to 0.284) |

For each country population weighted and adjusted for survey design estimate of the statistic characterizing the level and distribution of the respective malaria intervention coverage indictor is reported in each column. 95% confidence intervals are reported in the parentheses below the estimate. Q1 and Q5 denote respectively the lowest and highest asset-wealth quintiles. CIX was implemented with *conindex* command in Stata SE 14. SII was computed on individual data; estimates represent the difference in the predicted probabilities of the respective coverage indicator evaluated at highest and lowest values of the asset-wealth ranking variable (1 and 0) computed as marginal effects following probit estimation. For details of statistics evaluated refer to text and methodological guidance in [29]. *Data drawn from a subset of countries with DHS/MIS conducted after 2010 (country list and year of data collection are detailed in Additional file 1).

*CIX* Concentration Index, *SII* Slope Index of Inequality

**Table SA7 Distribution of children under five with fever that sought care at a formal provider in 2015***

| **Country** | **Total** | **Q1** | **Q5** | **Difference Q5-Q1** | **Ratio Q5:Q1** | **CIX** | **SII** |
| --- | --- | --- | --- | --- | --- | --- | --- |
| Angola | 0.557 (0.522 to 0.593) | 0.404 (0.310 to 0.498) | 0.767 (0.724 to 0.811) | 0.363 (0.260 to 0.467) | 1.899 (1.444 to 2.353) | 0.292 (0.204 to 0.379) | 0.405 (0.307 to 0.504) |
| Benin | 0.386 (0.355 to 0.418) | 0.276 (0.207 to 0.346) | 0.472 (0.407 to 0.538) | 0.196 (0.100 to 0.291) | 1.707 (1.217 to 2.197) | 0.141 (0.071 to 0.210) | 0.329 (0.165 to 0.493) |
| Burkina Faso | 0.586 (0.556 to 0.616) | 0.444 (0.384 to 0.504) | 0.620 (0.544 to 0.696) | 0.176 (0.079 to 0.273) | 1.396 (1.142 to 1.651) | 0.166 (0.089 to 0.243) | 0.255 (-0.034 to 0.544) |
| Burundi | 0.542 (0.512 to 0.573) | 0.560 (0.505 to 0.615) | 0.564 (0.484 to 0.643) | 0.004 (-0.095 to 0.102) | 1.006 (0.830 to 1.183) | 0.007 (-0.062 to 0.075) | 0.086 (-0.118 to 0.290) |
| Cameroon | 0.270 (0.243 to 0.298) | 0.115 (0.085 to 0.146) | 0.430 (0.373 to 0.487) | 0.315 (0.250 to 0.379) | 3.724 (2.630 to 4.819) | 0.261 (0.212 to 0.309) | 0.555 (0.453 to 0.656) |
| Chad | 0.231 (0.206 to 0.257) | 0.136 (0.098 to 0.173) | 0.369 (0.324 to 0.414) | 0.233 (0.174 to 0.292) | 2.719 (1.888 to 3.551) | 0.182 (0.138 to 0.225) | 0.571 (0.448 to 0.695) |
| Comoros | 0.447 (0.389 to 0.504) | 0.405 (0.293 to 0.516) | 0.495 (0.371 to 0.618) | 0.090 (-0.070 to 0.250) | 1.223 (0.785 to 1.661) | 0.117 (-0.001 to 0.234) | 0.255 (-0.006 to 0.516) |
| Congo | 0.466 (0.423 to 0.510) | 0.403 (0.361 to 0.444) | 0.610 (0.516 to 0.703) | 0.207 (0.104 to 0.310) | 1.515 (1.234 to 1.795) | 0.129 (0.056 to 0.201) | 0.244 (0.118 to 0.371) |
| Congo, Democratic Republic | 0.398 (0.373 to 0.424) | 0.374 (0.331 to 0.417) | 0.415 (0.354 to 0.477) | 0.041 (-0.034 to 0.116) | 1.110 (0.902 to 1.318) | 0.019 (-0.033 to 0.071) | 0.133 (-0.015 to 0.281) |
| Cote d’Ivoire | 0.338 (0.306 to 0.370) | 0.216 (0.145 to 0.287) | 0.514 (0.437 to 0.592) | 0.298 (0.192 to 0.404) | 2.378 (1.513 to 3.242) | 0.198 (0.123 to 0.273) | 0.489 (0.326 to 0.653) |
| Gabon | 0.502 (0.439 to 0.566) | 0.444 (0.378 to 0.511) | 0.579 (0.377 to 0.781) | 0.134 (-0.074 to 0.343) | 1.303 (0.820 to 1.785) | 0.068 (-0.067 to 0.202) | 0.153 (-0.139 to 0.444) |
| Ghana | 0.559 (0.511 to 0.607) | 0.551 (0.474 to 0.628) | 0.540 (0.411 to 0.669) | -0.011 (-0.161 to 0.139) | 0.980 (0.709 to 1.252) | 0.007 (-0.100 to 0.114) | -0.045 (-0.358 to 0.267) |
| Guinea | 0.334 (0.299 to 0.369) | 0.206 (0.152 to 0.261) | 0.595 (0.504 to 0.687) | 0.389 (0.282 to 0.495) | 2.882 (1.999 to 3.766) | 0.265 (0.198 to 0.332) | 0.564 (0.444 to 0.683) |
| Kenya | 0.656 (0.618 to 0.693) | 0.582 (0.507 to 0.658) | 0.738 (0.660 to 0.817) | 0.156 (0.045 to 0.267) | 1.268 (1.051 to 1.485) | 0.111 (0.032 to 0.190) | 0.300 (0.108 to 0.491) |
| Liberia | 0.574 (0.535 to 0.613) | 0.506 (0.454 to 0.559) | 0.622 (0.490 to 0.754) | 0.116 (-0.029 to 0.261) | 1.229 (0.932 to 1.526) | 0.099 (0.014 to 0.184) | 0.241 (0.032 to 0.449) |
| Madagascar | 0.521 (0.476 to 0.567) | 0.504 (0.436 to 0.573) | 0.562 (0.422 to 0.703) | 0.058 (-0.098 to 0.214) | 1.115 (0.798 to 1.431) | 0.061 (-0.041 to 0.163) | 0.197 (-0.176 to 0.570) |
| Malawi | 0.614 (0.593 to 0.634) | 0.602 (0.566 to 0.639) | 0.607 (0.557 to 0.657) | 0.005 (-0.055 to 0.064) | 1.008 (0.908 to 1.107) | 0.000 (-0.041 to 0.042) | -0.023 (-0.159 to 0.112) |
| Mali | 0.291 (0.247 to 0.335) | 0.181 (0.098 to 0.263) | 0.548 (0.472 to 0.623) | 0.367 (0.255 to 0.479) | 3.034 (1.583 to 4.485) | 0.224 (0.140 to 0.308) | 0.773 (0.678 to 0.867) |
| Mozambique | 0.587 (0.549 to 0.626) | 0.538 (0.465 to 0.610) | 0.687 (0.609 to 0.766) | 0.150 (0.043 to 0.257) | 1.278 (1.052 to 1.504) | 0.178 (0.105 to 0.252) | 0.328 (0.200 to 0.456) |
| Namibia | 0.586 (0.546 to 0.627) | 0.570 (0.492 to 0.649) | 0.568 (0.470 to 0.666) | -0.003 (-0.128 to 0.123) | 0.995 (0.775 to 1.215) | -0.002 (-0.092 to 0.088) | -0.021 (-0.184 to 0.142) |
| Niger | 0.508 (0.470 to 0.546) | 0.409 (0.331 to 0.487) | 0.616 (0.556 to 0.676) | 0.207 (0.109 to 0.305) | 1.505 (1.184 to 1.826) | 0.135 (0.062 to 0.208) | 0.359 (0.184 to 0.533) |
| Nigeria | 0.315 (0.290 to 0.339) | 0.252 (0.209 to 0.295) | 0.436 (0.374 to 0.499) | 0.184 (0.108 to 0.260) | 1.729 (1.345 to 2.114) | 0.137 (0.084 to 0.190) | 0.341 (0.223 to 0.459) |
| Rwanda | 0.492 (0.462 to 0.522) | 0.386 (0.331 to 0.440) | 0.624 (0.562 to 0.687) | 0.239 (0.156 to 0.322) | 1.619 (1.337 to 1.900) | 0.182 (0.120 to 0.243) | 0.465 (0.335 to 0.596) |
| Senegal | 0.458 (0.398 to 0.518) | 0.442 (0.357 to 0.528) | 0.564 (0.377 to 0.752) | 0.122 (-0.084 to 0.328) | 1.275 (0.785 to 1.765) | 0.072 (-0.086 to 0.229) | 0.130 (-0.172 to 0.432) |
| Sierra Leone | 0.656 (0.626 to 0.686) | 0.652 (0.600 to 0.704) | 0.564 (0.494 to 0.633) | -0.088 (-0.175 to -0.001) | 0.865 (0.738 to 0.992) | -0.034 (-0.096 to 0.029) | -0.230 (-0.456 to -0.005) |
| Tanzania | 0.660 (0.628 to 0.691) | 0.560 (0.498 to 0.621) | 0.795 (0.733 to 0.856) | 0.235 (0.147 to 0.323) | 1.420 (1.227 to 1.613) | 0.199 (0.134 to 0.265) | 0.478 (0.359 to 0.597) |
| Togo | 0.389 (0.351 to 0.427) | 0.362 (0.300 to 0.425) | 0.563 (0.484 to 0.643) | 0.201 (0.100 to 0.302) | 1.555 (1.208 to 1.901) | 0.130 (0.051 to 0.209) | 0.554 (0.366 to 0.743) |
| Uganda | 0.762 (0.724 to 0.801) | 0.701 (0.642 to 0.759) | 0.844 (0.768 to 0.920) | 0.143 (0.049 to 0.237) | 1.205 (1.059 to 1.350) | 0.123 (0.057 to 0.190) | 0.276 (0.120 to 0.432) |
| Zambia | 0.719 (0.696 to 0.742) | 0.686 (0.641 to 0.730) | 0.752 (0.687 to 0.818) | 0.066 (-0.013 to 0.145) | 1.096 (0.977 to 1.215) | 0.050 (-0.001 to 0.101) | 0.119 (0.008 to 0.231) |
| Zimbabwe | 0.451 (0.401 to 0.500) | 0.419 (0.334 to 0.504) | 0.605 (0.514 to 0.695) | 0.186 (0.061 to 0.310) | 1.443 (1.078 to 1.808) | 0.153 (0.055 to 0.252) | 0.374 (0.176 to 0.572) |

For each country population weighted and adjusted for survey design estimate of the statistic characterizing the level and distribution of the respective malaria intervention coverage indictor is reported in each column. 95% confidence intervals are reported in the parentheses below the estimate. Q1 and Q5 denote respectively the lowest and highest asset-wealth quintiles. CIX was implemented with *conindex* command in Stata SE 14. SII was computed on individual data; estimates represent the difference in the predicted probabilities of the respective coverage indicator evaluated at highest and lowest values of the asset-wealth ranking variable (1 and 0) computed as marginal effects following probit estimation. For details of statistics evaluated refer to text and methodological guidance in [29]. *Data drawn from a subset of countries with DHS/MIS conducted after 2010 (country list and year of data collection are detailed in Additional file 1).

*CIX* Concentration Index, *SII* Slope Index of Inequality

**Table SA8 Distribution of children under five with fever that were treated with an antimalarial medication in 2015***

| **Country** | **Total** | **Q1** | **Q5** | **Difference Q5-Q1** | **Ratio Q5:Q1** | **CIX** | **SII** |
| --- | --- | --- | --- | --- | --- | --- | --- |
| Angola | 0.283 (0.248 to 0.318) | 0.181 (0.072 to 0.290) | 0.519 (0.465 to 0.573) | 0.338 (0.216 to 0.460) | 2.865 (1.111 to 4.620) | 0.268 (0.163 to 0.373) | 0.420 (0.295 to 0.544) |
| Benin | 0.384 (0.350 to 0.417) | 0.267 (0.198 to 0.337) | 0.444 (0.361 to 0.527) | 0.176 (0.069 to 0.284) | 1.660 (1.132 to 2.188) | 0.104 (0.025 to 0.184) | 0.236 (0.035 to 0.438) |
| Burkina Faso | 0.492 (0.462 to 0.521) | 0.361 (0.313 to 0.409) | 0.563 (0.465 to 0.661) | 0.202 (0.093 to 0.311) | 1.558 (1.217 to 1.899) | 0.192 (0.125 to 0.260) | 0.422 (0.224 to 0.620) |
| Burundi | 0.255 (0.222 to 0.287) | 0.290 (0.243 to 0.338) | 0.244 (0.171 to 0.317) | -0.047 (-0.130 to 0.037) | 0.840 (0.564 to 1.115) | -0.038 (-0.102 to 0.027) | -0.158 (-0.337 to 0.021) |
| Cameroon | 0.231 (0.207 to 0.256) | 0.066 (0.040 to 0.093) | 0.317 (0.259 to 0.374) | 0.250 (0.187 to 0.314) | 4.781 (2.685 to 6.878) | 0.236 (0.192 to 0.281) | 0.481 (0.371 to 0.591) |
| Chad | 0.269 (0.235 to 0.303) | 0.226 (0.175 to 0.278) | 0.315 (0.266 to 0.365) | 0.089 (0.017 to 0.161) | 1.394 (1.007 to 1.780) | 0.065 (0.013 to 0.118) | 0.212 (0.031 to 0.392) |
| Comoros | 0.267 (0.219 to 0.316) | 0.164 (0.081 to 0.248) | 0.332 (0.192 to 0.471) | 0.167 (0.007 to 0.327) | 2.016 (0.706 to 3.325) | 0.108 (-0.008 to 0.223) | 0.264 (0.002 to 0.525) |
| Congo | 0.139 (0.118 to 0.159) | 0.122 (0.096 to 0.147) | 0.152 (0.085 to 0.219) | 0.030 (-0.042 to 0.102) | 1.247 (0.637 to 1.857) | 0.030 (-0.017 to 0.077) | 0.047 (-0.043 to 0.138) |
| Congo, Democratic Republic | 0.292 (0.268 to 0.316) | 0.225 (0.179 to 0.270) | 0.433 (0.372 to 0.494) | 0.208 (0.133 to 0.284) | 1.928 (1.455 to 2.401) | 0.156 (0.104 to 0.208) | 0.448 (0.319 to 0.577) |
| Cote d’Ivoire | 0.175 (0.146 to 0.204) | 0.103 (0.062 to 0.145) | 0.280 (0.210 to 0.350) | 0.177 (0.095 to 0.258) | 2.708 (1.432 to 3.985) | 0.128 (0.074 to 0.182) | 0.330 (0.150 to 0.509) |
| Gabon | 0.259 (0.207 to 0.312) | 0.189 (0.149 to 0.230) | 0.280 (0.169 to 0.392) | 0.091 (-0.025 to 0.207) | 1.480 (0.829 to 2.131) | 0.104 (0.033 to 0.175) | 0.201 (0.049 to 0.354) |
| Ghana | 0.485 (0.439 to 0.532) | 0.414 (0.336 to 0.492) | 0.529 (0.392 to 0.665) | 0.115 (-0.043 to 0.272) | 1.277 (0.869 to 1.686) | 0.089 (-0.022 to 0.200) | 0.223 (-0.089 to 0.536) |
| Guinea | 0.281 (0.250 to 0.312) | 0.224 (0.171 to 0.276) | 0.328 (0.252 to 0.403) | 0.104 (0.012 to 0.196) | 1.465 (0.985 to 1.946) | 0.108 (0.047 to 0.170) | 0.189 (0.057 to 0.320) |
| Kenya | 0.271 (0.228 to 0.314) | 0.197 (0.145 to 0.250) | 0.196 (0.132 to 0.261) | -0.001 (-0.084 to 0.082) | 0.996 (0.575 to 1.416) | 0.025 (-0.050 to 0.100) | 0.005 (-0.190 to 0.199) |
| Liberia | 0.557 (0.523 to 0.591) | 0.518 (0.470 to 0.567) | 0.575 (0.435 to 0.715) | 0.057 (-0.091 to 0.205) | 1.110 (0.820 to 1.399) | 0.034 (-0.052 to 0.121) | 0.083 (-0.147 to 0.313) |
| Madagascar | 0.101 (0.073 to 0.129) | 0.101 (0.048 to 0.155) | 0.027 (0.004 to 0.050) | -0.075 (-0.133 to -0.016) | 0.264 (-0.004 to 0.532) | -0.039 (-0.090 to 0.011) | -0.185 (-0.321 to -0.049) |
| Malawi | 0.376 (0.354 to 0.398) | 0.404 (0.364 to 0.445) | 0.251 (0.206 to 0.297) | -0.153 (-0.215 to -0.092) | 0.621 (0.492 to 0.751) | -0.093 (-0.138 to -0.048) | -0.316 (-0.416 to -0.216) |
| Mali | 0.226 (0.184 to 0.267) | 0.189 (0.109 to 0.268) | 0.284 (0.209 to 0.359) | 0.095 (-0.014 to 0.205) | 1.505 (0.756 to 2.254) | 0.070 (-0.005 to 0.145) | 0.270 (-0.018 to 0.558) |
| Mozambique | 0.299 (0.264 to 0.334) | 0.361 (0.275 to 0.447) | 0.171 (0.094 to 0.247) | -0.190 (-0.305 to -0.075) | 0.473 (0.233 to 0.713) | -0.100 (-0.179 to -0.021) | -0.246 (-0.363 to -0.128) |
| Namibia | 0.084 (0.059 to 0.110) | 0.120 (0.062 to 0.177) | 0.044 (0.002 to 0.086) | -0.076 (-0.147 to -0.005) | 0.366 (-0.028 to 0.759) | -0.030 (-0.082 to 0.021) | -0.060 (-0.142 to 0.023) |
| Niger | 0.192 (0.165 to 0.219) | 0.105 (0.063 to 0.147) | 0.309 (0.240 to 0.377) | 0.203 (0.123 to 0.284) | 2.934 (1.590 to 4.277) | 0.143 (0.088 to 0.198) | 0.579 (0.355 to 0.804) |
| Nigeria | 0.327 (0.303 to 0.352) | 0.221 (0.184 to 0.258) | 0.475 (0.406 to 0.544) | 0.254 (0.175 to 0.332) | 2.145 (1.670 to 2.620) | 0.217 (0.167 to 0.267) | 0.481 (0.368 to 0.594) |
| Rwanda | 0.112 (0.090 to 0.134) | 0.110 (0.076 to 0.144) | 0.061 (0.014 to 0.108) | -0.049 (-0.107 to 0.010) | 0.558 (0.094 to 1.021) | -0.036 (-0.075 to 0.003) | -0.151 (-0.226 to -0.076) |
| Senegal | 0.067 (0.027 to 0.107) | 0.050 (0.022 to 0.078) | 0.148 (0.007 to 0.289) | 0.098 (-0.047 to 0.242) | 2.956 (-0.321 to 6.233) | 0.080 (-0.022 to 0.181) | 0.176 (-0.060 to 0.412) |
| Sierra Leone | 0.483 (0.450 to 0.517) | 0.493 (0.429 to 0.556) | 0.441 (0.379 to 0.503) | -0.052 (-0.141 to 0.037) | 0.895 (0.724 to 1.066) | -0.006 (-0.074 to 0.062) | -0.088 (-0.316 to 0.141) |
| Tanzania | 0.511 (0.480 to 0.542) | 0.555 (0.487 to 0.623) | 0.432 (0.367 to 0.497) | -0.123 (-0.216 to -0.030) | 0.779 (0.629 to 0.928) | -0.121 (-0.186 to -0.055) | -0.329 (-0.497 to -0.161) |
| Togo | 0.183 (0.153 to 0.213) | 0.219 (0.155 to 0.283) | 0.210 (0.138 to 0.282) | -0.009 (-0.105 to 0.088) | 0.960 (0.526 to 1.394) | -0.016 (-0.087 to 0.054) | 0.030 (-0.200 to 0.260) |
| Uganda | 0.770 (0.739 to 0.800) | 0.754 (0.711 to 0.797) | 0.789 (0.711 to 0.868) | 0.035 (-0.057 to 0.126) | 1.046 (0.923 to 1.169) | 0.008 (-0.055 to 0.072) | -0.022 (-0.216 to 0.172) |
| Zambia | 0.398 (0.371 to 0.426) | 0.456 (0.408 to 0.504) | 0.234 (0.171 to 0.297) | -0.222 (-0.301 to -0.144) | 0.513 (0.366 to 0.660) | -0.184 (-0.237 to -0.132) | -0.408 (-0.493 to -0.324) |
| Zimbabwe | 0.010 (0.002 to 0.017) | 0.003 (-0.003 to 0.009) | 0.028 (-0.000 to 0.055) | 0.025 (-0.004 to 0.053) | 9.176 ( -11.110 to 29.462) | 0.017 (-0.000 to 0.034) | 0.079 (-0.049 to 0.207) |

For each country population weighted and adjusted for survey design estimate of the statistic characterizing the level and distribution of the respective malaria intervention coverage indictor is reported in each column. 95% confidence intervals are reported in the parentheses below the estimate. Q1 and Q5 denote respectively the lowest and highest asset-wealth quintiles. CIX was implemented with *conindex* command in Stata SE 14. SII was computed on individual data; estimates represent the difference in the predicted probabilities of the respective coverage indicator evaluated at highest and lowest values of the asset-wealth ranking variable (1 and 0) computed as marginal effects following probit estimation. For details of statistics evaluated refer to text and methodological guidance in [29]. *Data drawn from a subset of countries with DHS/MIS conducted after 2010 (country list and year of data collection are detailed in Additional file 1).

*CIX* Concentration Index, *SII* Slope Index of Inequality

**Table SA9 Distribution of children under five with fever that were treated with first-line antimalarial medication in 2015***

| **Country** | **Total** | **Q1** | **Q5** | **Difference Q5-Q1** | **Ratio Q5:Q1** | **CIX** | **SII** |
| --- | --- | --- | --- | --- | --- | --- | --- |
| Angola | 0.217 (0.188 to 0.245) | 0.082 (0.051 to 0.114) | 0.442 (0.385 to 0.500) | 0.360 (0.294 to 0.426) | 5.371 (3.202 to 7.539) | 0.275 (0.232 to 0.318) | 0.419 (0.346 to 0.492) |
| Benin | 0.123 (0.100 to 0.146) | 0.055 (0.023 to 0.087) | 0.143 (0.083 to 0.203) | 0.088 (0.020 to 0.156) | 2.598 (0.739 to 4.456) | 0.059 (0.005 to 0.113) | 0.130 (-0.028 to 0.287) |
| Burkina Faso | 0.137 (0.118 to 0.157) | 0.077 (0.056 to 0.099) | 0.173 (0.118 to 0.228) | 0.095 (0.036 to 0.155) | 2.231 (1.290 to 3.173) | 0.097 (0.055 to 0.139) | 0.260 (0.048 to 0.471) |
| Burundi | 0.180 (0.153 to 0.206) | 0.200 (0.157 to 0.243) | 0.159 (0.093 to 0.225) | -0.041 (-0.118 to 0.036) | 0.795 (0.431 to 1.159) | -0.026 (-0.076 to 0.024) | -0.181 (-0.312 to -0.051) |
| Cameroon | 0.061 (0.048 to 0.073) | 0.010 (0.002 to 0.019) | 0.111 (0.067 to 0.155) | 0.101 (0.056 to 0.146) | 10.762 (0.914 to 20.610) | 0.091 (0.065 to 0.118) | 0.272 (0.168 to 0.377) |
| Chad | 0.027 (0.019 to 0.035) | 0.017 (0.002 to 0.031) | 0.067 (0.041 to 0.093) | 0.050 (0.020 to 0.080) | 4.016 (0.205 to 7.827) | 0.041 (0.021 to 0.062) | 0.259 (0.117 to 0.400) |
| Comoros | 0.043 (0.010 to 0.076) | 0.003 (-0.003 to 0.009) | 0.095 (-0.032 to 0.222) | 0.092 (-0.035 to 0.219) | 31.843 ( -44.24 to 107.929) | 0.066 (-0.018 to 0.151) | 0.175 (-0.074 to 0.423) |
| Congo | 0.051 (0.036 to 0.066) | 0.014 (0.006 to 0.022) | 0.094 (0.044 to 0.145) | 0.080 (0.029 to 0.131) | 6.656 (1.500 to 11.811) | 0.064 (0.030 to 0.098) | 0.144 (0.057 to 0.231) |
| Congo, Democratic Republic | 0.056 (0.042 to 0.069) | 0.059 (0.030 to 0.088) | 0.069 (0.042 to 0.097) | 0.010 (-0.030 to 0.050) | 1.172 (0.427 to 1.917) | -0.004 (-0.030 to 0.023) | 0.023 (-0.051 to 0.096) |
| Cote d’Ivoire | 0.031 (0.017 to 0.044) | 0.018 (-0.004 to 0.041) | 0.052 (0.014 to 0.091) | 0.034 (-0.011 to 0.078) | 2.857 (-1.213 to 6.926) | 0.021 (-0.005 to 0.047) | 0.046 (-0.050 to 0.141) |
| Gabon | 0.088 (0.061 to 0.114) | 0.033 (0.016 to 0.051) | 0.148 (0.065 to 0.232) | 0.115 (0.030 to 0.200) | 4.456 (1.052 to 7.860) | 0.083 (0.026 to 0.139) | 0.194 (0.064 to 0.325) |
| Ghana | 0.379 (0.334 to 0.425) | 0.309 (0.236 to 0.383) | 0.419 (0.282 to 0.557) | 0.110 (-0.047 to 0.267) | 1.355 (0.803 to 1.908) | 0.076 (-0.035 to 0.188) | 0.212 (-0.101 to 0.526) |
| Guinea | 0.014 (0.007 to 0.020) | 0.019 (0.003 to 0.035) | 0.020 (0.001 to 0.038) | 0.001 (-0.024 to 0.025) | 1.030 (-0.279 to 2.339) | 0.010 (-0.008 to 0.028) | 0.033 (-0.017 to 0.083) |
| Kenya | 0.248 (0.207 to 0.290) | 0.165 (0.115 to 0.216) | 0.173 (0.110 to 0.236) | 0.008 (-0.074 to 0.089) | 1.046 (0.545 to 1.547) | 0.040 (-0.033 to 0.112) | 0.047 (-0.146 to 0.241) |
| Liberia | 0.239 (0.202 to 0.276) | 0.257 (0.210 to 0.305) | 0.204 (0.111 to 0.297) | -0.053 (-0.157 to 0.051) | 0.794 (0.406 to 1.182) | -0.059 (-0.134 to 0.017) | -0.143 (-0.311 to 0.026) |
| Madagascar | 0.017 (0.007 to 0.028) | 0.003 (-0.003 to 0.009) | 0.011 (-0.008 to 0.030) | 0.008 (-0.012 to 0.027) | 3.505 (-5.453 to 12.462) | 0.001 (-0.016 to 0.018) | -0.001 (-0.085 to 0.083) |
| Malawi | 0.345 (0.323 to 0.367) | 0.383 (0.343 to 0.424) | 0.217 (0.175 to 0.259) | -0.167 (-0.226 to -0.108) | 0.566 (0.440 to 0.692) | -0.105 (-0.148 to -0.061) | -0.345 (-0.420 to -0.270) |
| Mali | 0.043 (0.027 to 0.059) | 0.051 (0.009 to 0.093) | 0.054 (0.009 to 0.100) | 0.004 (-0.059 to 0.066) | 1.072 (-0.199 to 2.343) | -0.001 (-0.042 to 0.040) | 0.065 (-0.171 to 0.302) |
| Mozambique | 0.179 (0.147 to 0.211) | 0.220 (0.141 to 0.299) | 0.082 (0.040 to 0.124) | -0.138 (-0.227 to -0.049) | 0.373 (0.141 to 0.604) | -0.083 (-0.146 to -0.019) | -0.181 (-0.265 to -0.096) |
| Namibia | 0.038 (0.019 to 0.057) | 0.083 (0.034 to 0.132) | 0.000 (0.000 to 0.000) | -0.083 (-0.132 to -0.034) | 0.000 (0.000 to 0.000) | -0.047 (-0.081 to -0.013) | -0.073 (-0.119 to -0.026) |
| Niger | 0.153 (0.129 to 0.176) | 0.074 (0.041 to 0.107) | 0.250 (0.187 to 0.313) | 0.176 (0.105 to 0.248) | 3.379 (1.642 to 5.116) | 0.133 (0.083 to 0.182) | 0.546 (0.300 to 0.792) |
| Nigeria | 0.060 (0.049 to 0.070) | 0.036 (0.023 to 0.049) | 0.104 (0.074 to 0.134) | 0.068 (0.035 to 0.101) | 2.857 (1.546 to 4.168) | 0.058 (0.036 to 0.081) | 0.156 (0.094 to 0.217) |
| Rwanda | 0.112 (0.090 to 0.134) | 0.110 (0.076 to 0.144) | 0.061 (0.014 to 0.108) | -0.049 (-0.107 to 0.010) | 0.558 (0.094 to 1.021) | -0.036 (-0.075 to 0.003) | -0.151 (-0.226 to -0.076) |
| Senegal | 0.007 (0.002 to 0.012) | 0.022 (0.004 to 0.040) | 0.001 (-0.001 to 0.003) | -0.021 (-0.039 to -0.003) | 0.043 (-0.051 to 0.137) | -0.019 (-0.034 to -0.003) | -0.037 (-0.083 to 0.008) |
| Sierra Leone | 0.372 (0.335 to 0.409) | 0.412 (0.345 to 0.480) | 0.306 (0.256 to 0.357) | -0.106 (-0.191 to -0.021) | 0.743 (0.569 to 0.916) | -0.040 (-0.108 to 0.028) | -0.162 (-0.348 to 0.024) |
| Tanzania | 0.434 (0.402 to 0.466) | 0.507 (0.440 to 0.574) | 0.320 (0.256 to 0.385) | -0.187 (-0.280 to -0.094) | 0.631 (0.480 to 0.783) | -0.180 (-0.248 to -0.113) | -0.455 (-0.603 to -0.307) |
| Togo | 0.090 (0.072 to 0.109) | 0.073 (0.040 to 0.107) | 0.108 (0.058 to 0.157) | 0.034 (-0.026 to 0.094) | 1.462 (0.512 to 2.413) | 0.022 (-0.020 to 0.064) | 0.051 (-0.108 to 0.209) |
| Uganda | 0.667 (0.630 to 0.703) | 0.684 (0.633 to 0.736) | 0.574 (0.445 to 0.702) | -0.110 (-0.251 to 0.030) | 0.839 (0.638 to 1.039) | -0.064 (-0.149 to 0.022) | -0.294 (-0.568 to -0.021) |
| Zambia | 0.360 (0.333 to 0.387) | 0.428 (0.381 to 0.476) | 0.172 (0.113 to 0.231) | -0.257 (-0.332 to -0.181) | 0.401 (0.257 to 0.546) | -0.209 (-0.260 to -0.158) | -0.428 (-0.495 to -0.361) |
| Zimbabwe | 0.004 (-0.001 to 0.009) | 0.003 (-0.003 to 0.009) | 0.002 (-0.002 to 0.007) | -0.001 (-0.008 to 0.007) | 0.733 (-1.308 to 2.774) | -0.000 (-0.005 to 0.005) | -0.003 (-0.013 to 0.007) |

For each country population weighted and adjusted for survey design estimate of the statistic characterizing the level and distribution of the respective malaria intervention coverage indictor is reported in each column. 95% confidence intervals are reported in the parentheses below the estimate. Q1 and Q5 denote respectively the lowest and highest asset-wealth quintiles. CIX was implemented with *conindex* command in Stata SE 14. SII was computed on individual data; estimates represent the difference in the predicted probabilities of the respective coverage indicator evaluated at highest and lowest values of the asset-wealth ranking variable (1 and 0) computed as marginal effects following probit estimation. For details of statistics evaluated refer to text and methodological guidance in [29]. *Data drawn from a subset of countries with DHS/MIS conducted after 2010 (country list and year of data collection are detailed in Additional file 1).

*CIX* Concentration Index, *SII* Slope Index of Inequality
