# Supplementary material for: State of inequality in malaria intervention coverage in sub-Saharan African countries
Source: BMC Med. 2017 Oct 18;15:185. doi: 10.1186/s12916-017-0948-8 (PMC5646111; doi:10.1186/s12916-017-0948-8)
Supplement: Supplementary file 3 — Plots of 2015* level and degree of inequality for each of the malaria intervention coverage indicators including ACTs (Figure SA1-SA2). (DOCX 84 kb) [file 12916_2017_948_MOESM3_ESM.docx]

**Additional file 3**

**Fig. SA1 Level and degree of asset-wealth inequality in distribution of malaria intervention coverage indicators in Sub-Saharan African countries in 2015***

For each country concentration index (CIX) of the indicator is plotted against population mean; whiskers denote the 95% confidence interval of the estimate. Country marker size is weighted with population size. Marker color code changing from bright blue to bright red refers to country mean malaria prevalence based on 2015 MAP estimates (corresponding values are given in appendix table S2). ***A*** illustrates CIX and country mean for proportion of households with at least one ITN for every two person in the house. ***B*** illustrates CIX and country mean for proportion of population that slept under an ITN the night prior to the survey. ***C*** illustrates CIX and country mean for proportion of households residing in dwellings that have been sprayed within the last 12 months. ***D*** illustrates CIX and country mean and CIX for proportion of women that received at least 3 doses of SP at an ANC visit during their most recent pregnancy. ***E*** illustrates CIX and country mean for proportion of children under the age of five with fever sought care at a formal provider. ***F*** illustrates CIX and country mean for proportion of children under the age of five with fever that were treated with an antimalarial medication. ^*^Data drawn from a subset of countries with DHS/MIS conducted after 2010 (country list and year of data collection are detailed in Additional file 1).

*CIX* Concentration Index, *MAP* Malaria Atlas Project, *DHS* Demographic and Health Survey, *MIS* Malaria Indicator Survey

***C***

***B***

***A***

|  |  |  |
| --- | --- | --- |
|   ***D*** |   ***E*** |   ***F*** |

**Fig. SA2 Distribution of first-line antimalarial medication across asset-wealth index among children under five with fever in Sub-Saharan African countries in 2015***

*A* country concentration index (CIX) for receipt of first-line antimalarial medication for fever among children under the age of five is plotted against population mean. *B* country CIX for antimalarial treatment is plotted against CIX for receipt of country first-line antimalarial medication for children under the age of five with fever. Whiskers denote the 95% confidence interval of the estimate. Country marker size is weighted with population size. Marker color code changing from bright blue to bright red refers to country mean malaria prevalence based on 2015 MAP estimates (corresponding values are given in Additional file 1). *Data drawn from a subset of countries with DHS/MIS conducted after 2010 (country list and year of data collection are detailed in Additional file 1).

*CIX* Concentration Index, *MAP* Malaria Atlas Project, *DHS* Demographic and Health Survey, *MIS* Malaria Indicator Survey

***A***

***B***
