# Supplementary material for: State of inequality in malaria intervention coverage in sub-Saharan African countries
Source: BMC Med. 2017 Oct 18;15:185. doi: 10.1186/s12916-017-0948-8 (PMC5646111; doi:10.1186/s12916-017-0948-8)
Supplement: Supplementary file 4 — Plots of excess change and change in each of the malaria intervention coverage indicators from 2005 to 2015* (Additional file 2: Figure SA2). (DOCX 65 kb) [file 12916_2017_948_MOESM4_ESM.docx]

**Additional file 4**

**Fig. SA3 Changes in distribution of malaria intervention coverage indicators by asset-wealth in Sub-Saharan African countries from 2005 to 2015***

***A*** illustrates for each country the difference in average annual change in the proportion of households with at least one ITN for every two person (defacto) in the house in the lowest asset-wealth quintile and that of the highest (annual absolute excess change) against average annual change in the population (percentage points). ***B*** illustrates for each country the difference in average annual change in the proportion of population that slept under an ITN the night prior to the survey in the lowest asset-wealth quintile and that of the highest (annual absolute excess change) against average annual change in the population (percentage points). ***C*** illustrates for each country the difference in average annual change in the proportion of households in dwelling that have been sprayed within the last 12 months in the lowest asset-wealth quintile and that of the highest (annual absolute excess change) against average annual change in the population (percentage points). ***D*** illustrates for each country the difference in average annual change in the proportion of women that received at least 3 doses of SP at an ANC visit during their most recent pregnancy in the lowest asset-wealth quintile and that of the highest (annual absolute excess change) against average annual change in the population (percentage points). ***E*** illustrates for each country the difference in average annual change in the proportion of children under the age of five with fever sought care at a formal provider (2 weeks recall) in the lowest asset-wealth quintile and that of the highest (annual absolute excess change) against average annual change in the population (percentage points). ***F*** illustrates for each country the difference in average annual change in the proportion of children under the age of five with fever that were treated with an antimalarial medication in the lowest asset-wealth quintile and that of the highest (annual absolute excess change) against average annual change in the population (percentage points). ^*^Data drawn from a subset of countries with repeated DHS/MIS conducted between 2005 and 2015 (country list, ISO3 code, and years of data collection detailed in Additional file 1).

*DHS* Demographic and Health Surveys, *MIS* Malaria Indicator Survey

| ***B***  ***A***   |  | ***C***   |
| --- | --- | --- |
| ***D***   | ***E***   | ***F***   |

**Additional file 5**

**Table SA10 Distribution of malaria prevalence across asset-wealth quintiles in Sub-Saharan African countries in 2015***

| **Country** | **Total** | **Q1** | **Q5** | **Difference**  **Q5-Q1** | **Ratio**  **Q5:Q1** | **CIX** | **SII** |
| --- | --- | --- | --- | --- | --- | --- | --- |
| Benin | 0.135 (0.097 to 0.173) | 0.213 (0.140 to 0.285) | 0.015 (0.000 to 0.029) | -0.198 (-0.272 to -0.124) | 0.069 (-0.002 to 0.140) | -0.192 (-0.254 to -0.130) | -0.271 (-0.346 to -0.197) |
| Burkina Faso | 0.248 (0.229 to 0.267) | 0.389 (0.344 to 0.433) | 0.049 (0.030 to 0.068) | -0.340 (-0.388 to -0.292) | 0.126 (0.075 to 0.177) | -0.273 (-0.311 to -0.234) | -0.719 (-0.784 to -0.654) |
| Burundi | 0.614 (0.590 to 0.638) | 0.666 (0.629 to 0.703) | 0.261 (0.197 to 0.325) | -0.404 (-0.477 to -0.331) | 0.393 (0.295 to 0.491) | -0.267 (-0.316 to -0.218) | -0.833 (-0.880 to -0.786) |
| Congo, Democratic Republic | 0.128 (0.101 to 0.155) | 0.197 (0.146 to 0.248) | 0.048 (0.026 to 0.071) | -0.149 (-0.201 to -0.096) | 0.245 (0.123 to 0.367) | -0.109 (-0.147 to -0.071) | -0.590 (-0.783 to -0.396) |
| Cote d’Ivoire | 0.308 (0.278 to 0.338) | 0.333 (0.292 to 0.375) | 0.146 (0.111 to 0.182) | -0.187 (-0.242 to -0.132) | 0.438 (0.319 to 0.558) | -0.118 (-0.169 to -0.067) | -0.360 (-0.431 to -0.290) |
| Ghana | 0.415 (0.384 to 0.446) | 0.573 (0.521 to 0.625) | 0.122 (0.086 to 0.158) | -0.451 (-0.514 to -0.389) | 0.213 (0.148 to 0.278) | -0.372 (-0.430 to -0.315) | -0.677 (-0.730 to -0.624) |
| Guinea | 0.364 (0.331 to 0.396) | 0.600 (0.533 to 0.668) | 0.060 (0.020 to 0.100) | -0.540 (-0.619 to -0.462) | 0.100 (0.032 to 0.168) | -0.490 (-0.550 to -0.431) | -0.976 (-0.998 to -0.954) |
| Kenya | 0.469 (0.433 to 0.505) | 0.653 (0.601 to 0.704) | 0.061 (0.024 to 0.097) | -0.592 (-0.655 to -0.528) | 0.093 (0.037 to 0.150) | -0.415 (-0.469 to -0.360) | -0.889 (-0.942 to -0.837) |
| Madagascar | 0.091 (0.070 to 0.111) | 0.096 (0.060 to 0.132) | 0.019 (-0.004 to 0.042) | -0.077 (-0.120 to -0.034) | 0.197 (-0.056 to 0.449) | -0.070 (-0.107 to -0.032) | -0.172 (-0.270 to -0.075) |
| Mali | 0.051 (0.032 to 0.070) | 0.109 (0.069 to 0.150) | 0.001 (-0.000 to 0.002) | -0.108 (-0.149 to -0.068) | 0.009 (-0.002 to 0.021) | -0.088 (-0.120 to -0.056) | -0.483 (-0.657 to -0.309) |
| Mozambique | 0.472 (0.443 to 0.502) | 0.640 (0.597 to 0.683) | 0.103 (0.075 to 0.130) | -0.538 (-0.590 to -0.485) | 0.160 (0.116 to 0.205) | -0.413 (-0.461 to -0.366) | -0.688 (-0.722 to -0.655) |
| Rwanda | 0.383 (0.352 to 0.414) | 0.550 (0.495 to 0.606) | 0.056 (0.035 to 0.078) | -0.494 (-0.552 to -0.436) | 0.102 (0.063 to 0.142) | -0.384 (-0.435 to -0.333) | -0.601 (-0.646 to -0.557) |
| Senegal | 0.078 (0.065 to 0.092) | 0.127 (0.100 to 0.154) | 0.013 (0.001 to 0.025) | -0.113 (-0.142 to -0.084) | 0.106 (0.010 to 0.202) | -0.094 (-0.119 to -0.068) | -0.242 (-0.316 to -0.168) |
| Tanzania | 0.011 (0.005 to 0.017) | 0.031 (0.013 to 0.049) | 0.000 (-0.000 to 0.001) | -0.031 (-0.049 to -0.013) | 0.005 (-0.005 to 0.015) | -0.027 (-0.042 to -0.013) | -0.076 (-0.126 to -0.026) |
| Togo | 0.145 (0.125 to 0.164) | 0.226 (0.186 to 0.266) | 0.010 (0.003 to 0.018) | -0.216 (-0.256 to -0.175) | 0.046 (0.013 to 0.078) | -0.189 (-0.224 to -0.153) | -0.543 (-0.629 to -0.457) |
| Uganda | 0.380 (0.348 to 0.412) | 0.515 (0.458 to 0.573) | 0.095 (0.062 to 0.127) | -0.420 (-0.487 to -0.354) | 0.184 (0.118 to 0.250) | -0.380 (-0.435 to -0.326) | -0.770 (-0.829 to -0.711) |

RDT confirmed malaria prevalence in children aged 6 to 59 months assessed in a representative sub-sample of the DHS/MIS surveyed population. 95% confidence intervals are reported in the parentheses below the estimate. Q1 and Q5 denote respectively the lowest and highest asset-wealth quintiles. CIX was implemented with conindex command in Stata SE 14. SII was computed on individual data; estimates represent the difference in the predicted probabilities of the respective coverage indicator evaluated at highest and lowest values of the asset-wealth ranking variable (1 and 0) computed as marginal effects following probit estimation. For details of statistics evaluated refer to text and methodological guidance in [29]. ^*^Data drawn from a subset of countries with DHS/MIS conducted after 2010 (year of data collection detailed in Additional file 1).

*RDT* Rapid Diagnostic Test*, CIX* Concentration Index, *SII* Slope Index of Inequality, *DHS* Demographic and Health Survey, *MIS* Malaria Indicator Survey
