# Supplementary material for: State of inequality in malaria intervention coverage in sub-Saharan African countries
Source: BMC Med. 2017 Oct 18;15:185. doi: 10.1186/s12916-017-0948-8 (PMC5646111; doi:10.1186/s12916-017-0948-8)
Supplement: Supplementary file 5 — 2015* malaria parasite prevalence estimates according to rapid diagnostic tests based on country DHS/MIS surveys. (DOC 45 kb) [file 12916_2017_948_MOESM5_ESM.doc]

**Additional file 5**

**Table SA10 Distribution of malaria prevalence across asset-wealth quintiles in Sub-Saharan African countries in 2015***

| **Country** | **Total** | **Q1** | **Q5** | **Difference**  **Q5-Q1** | **Ratio**  **Q5:Q1** | **CIX** |
| --- | --- | --- | --- | --- | --- | --- |
| Benin | 0.135 (0.097 to 0.173) | 0.213 (0.140 to 0.285) | 0.015 (0.000 to 0.029) | -0.198 (-0.272 to -0.124) | 0.069 (-0.002 to 0.140) | -0.192 (-0.254 to -0.130) |
| Burkina Faso | 0.248 (0.229 to 0.267) | 0.389 (0.344 to 0.433) | 0.049 (0.030 to 0.068) | -0.340 (-0.388 to -0.292) | 0.126 (0.075 to 0.177) | -0.273 (-0.311 to -0.234) |
| Burundi | 0.614 (0.590 to 0.638) | 0.666 (0.629 to 0.703) | 0.261 (0.197 to 0.325) | -0.404 (-0.477 to -0.331) | 0.393 (0.295 to 0.491) | -0.267 (-0.316 to -0.218) |
| Congo, Democratic Republic | 0.128 (0.101 to 0.155) | 0.197 (0.146 to 0.248) | 0.048 (0.026 to 0.071) | -0.149 (-0.201 to -0.096) | 0.245 (0.123 to 0.367) | -0.109 (-0.147 to -0.071) |
| Cote d’Ivoire | 0.308 (0.278 to 0.338) | 0.333 (0.292 to 0.375) | 0.146 (0.111 to 0.182) | -0.187 (-0.242 to -0.132) | 0.438 (0.319 to 0.558) | -0.118 (-0.169 to -0.067) |
| Ghana | 0.415 (0.384 to 0.446) | 0.573 (0.521 to 0.625) | 0.122 (0.086 to 0.158) | -0.451 (-0.514 to -0.389) | 0.213 (0.148 to 0.278) | -0.372 (-0.430 to -0.315) |
| Guinea | 0.364 (0.331 to 0.396) | 0.600 (0.533 to 0.668) | 0.060 (0.020 to 0.100) | -0.540 (-0.619 to -0.462) | 0.100 (0.032 to 0.168) | -0.490 (-0.550 to -0.431) |
| Kenya | 0.469 (0.433 to 0.505) | 0.653 (0.601 to 0.704) | 0.061 (0.024 to 0.097) | -0.592 (-0.655 to -0.528) | 0.093 (0.037 to 0.150) | -0.415 (-0.469 to -0.360) |
| Madagascar | 0.091 (0.070 to 0.111) | 0.096 (0.060 to 0.132) | 0.019 (-0.004 to 0.042) | -0.077 (-0.120 to -0.034) | 0.197 (-0.056 to 0.449) | -0.070 (-0.107 to -0.032) |
| Mali | 0.051 (0.032 to 0.070) | 0.109 (0.069 to 0.150) | 0.001 (-0.000 to 0.002) | -0.108 (-0.149 to -0.068) | 0.009 (-0.002 to 0.021) | -0.088 (-0.120 to -0.056) |
| Mozambique | 0.472 (0.443 to 0.502) | 0.640 (0.597 to 0.683) | 0.103 (0.075 to 0.130) | -0.538 (-0.590 to -0.485) | 0.160 (0.116 to 0.205) | -0.413 (-0.461 to -0.366) |
| Rwanda | 0.383 (0.352 to 0.414) | 0.550 (0.495 to 0.606) | 0.056 (0.035 to 0.078) | -0.494 (-0.552 to -0.436) | 0.102 (0.063 to 0.142) | -0.384 (-0.435 to -0.333) |
| Senegal | 0.078 (0.065 to 0.092) | 0.127 (0.100 to 0.154) | 0.013 (0.001 to 0.025) | -0.113 (-0.142 to -0.084) | 0.106 (0.010 to 0.202) | -0.094 (-0.119 to -0.068) |
| Tanzania | 0.011 (0.005 to 0.017) | 0.031 (0.013 to 0.049) | 0.000 (-0.000 to 0.001) | -0.031 (-0.049 to -0.013) | 0.005 (-0.005 to 0.015) | -0.027 (-0.042 to -0.013) |
| Togo | 0.145 (0.125 to 0.164) | 0.226 (0.186 to 0.266) | 0.010 (0.003 to 0.018) | -0.216 (-0.256 to -0.175) | 0.046 (0.013 to 0.078) | -0.189 (-0.224 to -0.153) |
| Uganda | 0.380 (0.348 to 0.412) | 0.515 (0.458 to 0.573) | 0.095 (0.062 to 0.127) | -0.420 (-0.487 to -0.354) | 0.184 (0.118 to 0.250) | -0.380 (-0.435 to -0.326) |

RDT confirmed malaria prevalence in children aged 6 to 59 months assessed in a representative sub-sample of the DHS/MIS surveyed population. 95% confidence intervals are reported in the parentheses below the estimate. Q1 and Q5 denote respectively the lowest and highest asset-wealth quintiles. CIX was implemented with conindex command in Stata SE 14. SII was computed on individual data; estimates represent the difference in the predicted probabilities of the respective coverage indicator evaluated at highest and lowest values of the asset-wealth ranking variable (1 and 0) computed as marginal effects following probit estimation. For details of statistics evaluated refer to text and methodological guidance in [29]. ^*^Data drawn from a subset of countries with DHS/MIS conducted after 2010 (year of data collection detailed in Additional file 1).

*RDT* Rapid Diagnostic Test*, CIX* Concentration Index, *SII* Slope Index of Inequality, *DHS* Demographic and Health Survey, *MIS* Malaria Indicator Survey
